# Bioinspired Layered-Gradient Nanocomposites for Intelligent Electromagnetic Skins with GHz-THz Wave Absorption, Shielding, and Solvent-Driven Actuation

**DOI:** 10.1007/s40820-026-02202-y

**Published:** 2026-04-24

**Authors:** Xianyuan Liu, Yang Zhao, Yali Zhang, Xuechun Cui, Ying Xue, Xianyong Lu, Junwei Gu

**Affiliations:** 1 State Key Laboratory of Bioinspired Interfacial Materials Science, School of Chemistry, Beihang University, Beijing, 100191 People’s Republic of China; 2 Shaanxi Key Laboratory of Macromolecular Science and Technology, School of Chemistry and Chemical Engineering, Northwestern Polytechnical University, Xi’an, 710072 People’s Republic of China

**Keywords:** Electromagnetic interference shielding, Electromagnetic wave absorption, Gradient architecture, Terahertz, Intelligent actuation

## Abstract

The development of intelligent electromagnetic skins demands scalable films integrating gigahertz (GHz)-terahertz (THz) wave absorption, electromagnetic interference (EMI) shielding, and programmable actuation. Here, we report a bioinspired bamboo-like layered-gradient system fabricated via scalable vacuum filtration, in which two types of films are constructed with distinct functionalities: a low-poly(3,4-ethylenedioxythiophene) (PEDOT) content film for microwave absorption and a high PEDOT content film for conductive network-enabled EMI shielding.The films precisely assemble Al-Fe_3_O_4_ nanosheets, aramid nanofibers, and PEDOT into an asymmetric architecture. A monotonic through-thickness gradient in composition creates a tailored impedance profile and strong anisotropy while minimizing conductive filler content. This design achieves effective microwave absorption at low PEDOT loading (minimum reflection loss: − 56.6 dB at 2.2 mm in the X-band). With increased PEDOT content, a percolative network is constructed, enabling Joule heating (233 °C at 20 V) and efficient EMI shielding (42.0 dB in the GHz band and 57.8 dB in the THz band). Beyond electromagnetic performance, the gradient architecture enables programmable, ethanol-triggered anisotropic actuation via differential swelling. The films also exhibit excellent thermal stability, mechanical robustness, and flexibility, ensuring reliability in harsh environments. Collectively, this gradient architecture provides a scalable platform for intelligent electromagnetic skins integrate magnetic-dielectric coupling, conductive network tuning, and stimuli-responsive actuation.

**Supplementary Information:**

The online version contains supplementary material available at 10.1007/s40820-026-02202-y.

## Highlights

- A bamboo-inspired Al-Fe_3_O_4_/aramid nanofibers/poly(3,4-ethylenedioxythiophene) nanocomposite film with a through-thickness gradient architecture was constructed via scalable vacuum-assisted filtration.
- Gradient-regulated magnetic-dielectric coupling and percolative conductive networks enabled strong impedance matching and efficient electromagnetic attenuation across the gigahertz (GHz)-terahertz (THz) bands.
- An outstanding reflection loss (− 56.6 dB at 2.2 mm), broadband absorption (3.5 GHz), high electromagnetic interference shielding effectiveness (42.0 dB in GHz and 57.8 dB in THz), and reduced radar cross section were achieved.

**Supplementary Information:**

The online version contains supplementary material available at 10.1007/s40820-026-02202-y.

## Graphical abstract

**Figure:**
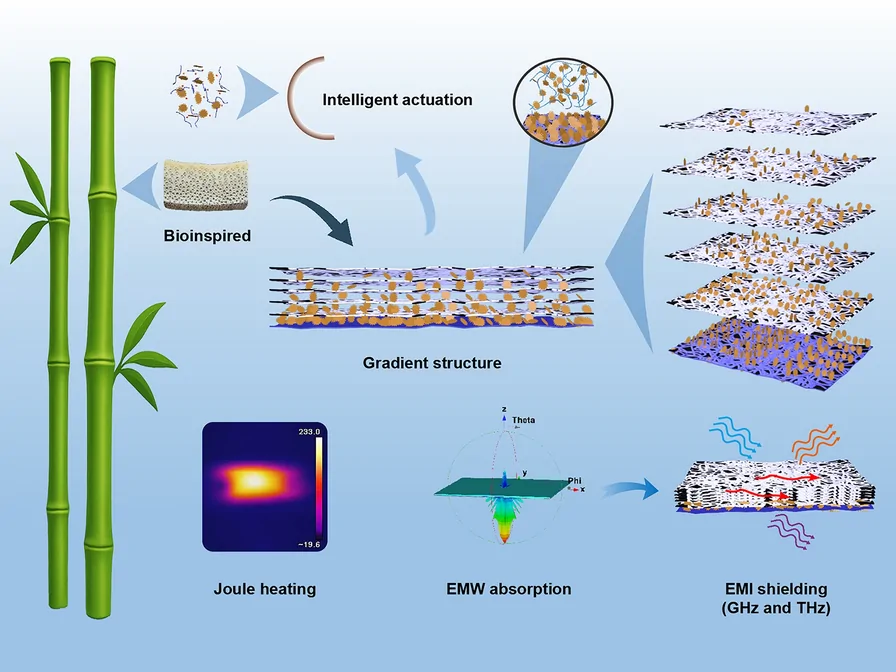

## Introduction

The rapid expansion of aerospace, electronic communications, artificial intelligence, and wearable technologies has triggered a proliferation of electronic devices, exacerbating the critical issue of electromagnetic pollution. Electromagnetic wave (EMW) not only interfere with precision instrumentation and pose risks of information leakage, but also raise growing concerns regarding human health [1]. To mitigate these challenges, there is an urgent demand for high-performance materials that offer both effective electromagnetic interference (EMI) shielding and efficient EMW absorption, particularly within the gigahertz (GHz) and terahertz (THz) frequency bands that are foundational to emerging technologies such as next-generation 6G communications [2]. Owing to their high frequencies, GHz-THz EMW are especially vulnerable to external perturbations, leading to signal degradation, data loss, and compromised security [3]. Consequently, the development of materials that integrate efficient EMW absorption with robust EMI shielding across these bands, while maintaining reliable functionality under harsh and dynamically varying service conditions, has become an urgent imperative [4, 5].

Polymer-based composites have attracted sustained interest for EMW management owing to their design flexibility and processability [6–8]. Among them, aramid nanofibers (ANFs), produced by disrupting hydrogen bonds in aramid microfibers followed by reassembly, offer a compelling platform due to their exceptional tensile strength, thermal stability, chemical resistance, and high specific surface area [9–11]. The abundance of polar surface functional groups (e.g., –C=O and –NH–) on ANFs facilitates robust interfacial adhesion with diverse functional fillers, thereby enhancing the structural integrity and broadening the multifunctional potential of the resulting composites [12, 13]. Meanwhile, the conductive polymer poly(3,4-ethylenedioxythiophene) (PEDOT) is valued for its high electrical conductivity, low density, flexibility, and environmental stability, making it an effective booster of EMI performance [14, 15]. By modulating interfacial charge transport and constructing heterogeneous relaxation interfaces, PEDOT intensifies dielectric losses and improves impedance matching. Incorporation of magnetic nanosheets into an ANFs-PEDOT matrix further introduces magnetic-dielectric synergy and additional interfacial heterogeneity, amplifying electromagnetic attenuation through concurrent magnetic loss and dielectric dissipation.

Beyond compositional design, bioinspired structural engineering offers powerful levers to optimize the performance of multifunctional composites by enabling the rational design of sustainable, recyclable, and high-performance materials and facilitating accurate electromagnetic parameter characterization for complex composite structures [16–20]. Bamboo, for example, exhibits a radially graded distribution of load-bearing cellulose fibers within a lignin–hemicellulose matrix, concentrating reinforcement near the outer wall to achieve superior stiffness and toughness [21]. At the molecular scale, an extensive hydrogen-bonded network confers remarkable resilience and damage tolerance. Translating such hierarchical and gradient principles into synthetic material systems enables deliberate through-thickness distribution of functional constituents such as magnetic nanosheets and conductive polymers within a fibrous ANF matrix. The resulting anisotropic architectures enable spatially resolved functionalities. Local enrichment of magnetic and conductive components can be tailored to enhance charge transport and EMW attenuation, while compositional asymmetry across the cross section can be harnessed to achieve stimuli-responsive actuation. Moreover, the multitude of internal interfaces promotes repeated scattering and reflection of incident waves, thereby significantly elevating EMI shielding effectiveness (SE) [4, 22–24]. Consequently, layered-gradient architectures represent a highly promising pathway for integrating mechanical robustness, thermal stability, efficient electromagnetic regulation, and intelligent actuation within a unified material platform.

Motivated by these insights, we report the rational design and fabrication of a multifunctional nanocomposite film. This material comprises sawtooth-edged Al-Fe_3_O_4_ nanosheets, ANFs, and PEDOT, assembled into a bamboo-inspired, through-thickness layered-gradient film via a scalable vacuum-assisted filtration process. Spatial control over the distributions of sawtooth-edged Al-Fe_3_O_4_ nanosheet and PEDOT across the cross section enables precise tuning of composite properties. By strategically modulating the PEDOT content relative to the magnetic framework, we realize synergistic GHz-THz EMI shielding and high-efficiency EMW absorption. The composite film simultaneously exhibits rapid Joule heating, programmable ethanol-responsive anisotropic actuation, and excellent thermal and mechanical resilience. This gradient architecture design strategy establishes a versatile and scalable platform for next-generation EMW management and paves the way for advanced intelligent multifunctional devices.

## Experimental Section

### Materials

Kevlar 49 fibers were obtained from DuPont (Wilmington, DE, USA). Aluminum chloride (AlCl_3_), ferric nitrate nonahydrate (Fe(NO_3_)_3_·9H_2_O), sodium oxalate, triethylamine, dodecylbenzenesulfonic acid (DBSA) (soft type, 90% mixture), potassium hydroxide (KOH), and absolute ethanol were purchased from Shanghai Macklin Biochemical Co., Ltd (Shanghai, China). Dimethyl sulfoxide (DMSO) was supplied by Shanghai Aladdin Biochemical Technology Co., Ltd (Shanghai, China). An aqueous poly(3,4-ethylenedioxythiophene): poly(styrene sulfonate) (1:2.5 PEDOT:PSS w/w ratio) dispersion (Clevios pH1000, 1.4 wt%) was procured from Heraeus Deutschland GmbH & Co. KG. Unless otherwise stated, all reagents were used as received.

### Preparation of Al-Fe_3_O_4_ Nanosheets

Al-Fe_3_O_4_ nanosheets were synthesized following our previous reported protocol [25]. Briefly, 0.100 g of AlCl_3_ and 1.212 g of Fe(NO_3_)_3_·9H_2_O were dissolved in 30 mL of deionized water under magnetic stirring to afford a clear solution. Triethylamine (4.5 mL) was then added dropwise, and the mixture was stirred for an additional 40 min. The precursor solution was transferred to a 100 mL Teflon-lined autoclave and heated at 160 °C for 24 h. The resulting red precipitate was collected by centrifugation, thoroughly washed with DI water, and dried to yield Al-doped α-Fe_2_O_3_ (Al-α-Fe_2_O_3_) nanosheets. The Al-α-Fe_2_O_3_ was subsequently redispersed in water to give a 12 mg mL^−1^ suspension.

To convert Al-*α*-Fe_2_O_3_ to Al-doped Fe_3_O_4_, 5.5 mL of the above suspension, 0.010 g of sodium dodecyl sulfate (SDS), and 0.121 g of Fe(NO_3_)_3_·9H_2_O were ultrasonicated at 100 W for 20 min. The resulting dispersion was centrifuged at 3500 rpm for 3 min to obtain a precipitate, which, along with 2.0 g sodium oxalate, was placed in separate ceramic boats inside a tube furnace (sodium oxalate upstream). Annealing was carried out at 600 °C for 5 h under Ar atmosphere (heating rate of 5 °C min^−1^) to afford Al-doped Fe_3_O_4_ (Al-Fe_3_O_4_) nanosheets.

### Preparation of ANFs Dispersion

The ANFs solution was prepared following a previous reported method [26]. Kevlar fibers (0.10 g) and KOH (0.15 g) were added to DMSO (10 mL) and stirred for 7 days to afford a dark-red solution of para-aramid nanofibers solution. Subsequently, additional DMSO (45 mL) and deionized (DI) water (160 mL) were gradually introduced under continuous stirring, inducing phase separation of the polymer solution and leading to the precipitation of ANFs. The mixture was further stirred for 24 h to yield a stable ANFs dispersion. The resulting ANFs dispersion has a solid content of approximately 0.465 mg mL^−1^.

### Preparation of Modified PEDOT Solution

To enhance conductivity, PEDOT:PSS was modified by synergistic secondary doping and partial PSS removal. Clevios PH1000 (12 mL) was mixed with DMSO (0.720 mL) and DBSA (0.096 g), followed by ultrasonication at 100 W for 20 min to obtain the modified PEDOT solution.

### Preparation of Al-Fe_3_O_4_/ANFs/PEDOT Composites

For the preparation of the gradient composite, 0.100 g of Al-Fe_3_O_4_ nanosheets and 0.004 mL of the modified PEDOT solution were added to the ANFs dispersion and ultrasonicated at 100 W for 20 min to generate a homogeneous suspension. The resulting mixture was then subjected to vacuum-assisted filtration to afford a free-standing the Al-Fe_3_O_4_/ANFs/PEDOT film. Increasing the volume of the modified PEDOT solution to 8 mL and 12 mL yielded Al-Fe_3_O_4_/ANFs/PEDOT_2_ and Al-Fe_3_O_4_/ANFs/PEDOT_3_ films, respectively.

### Characterization

The morphologies of the powders and composite films were examined by SEM (JEOL JSM-7500F, Japan; TESCAN MIRA LMS, Czech Republic). Thermogravimetric analysis (TGA) was performed on a Netzsch STA449F5 from 25 to 800 °C at 10 °C min^−1^ under N_2_. Tensile properties were measured using an Instron 5565A universal testing machine at a crosshead speed of at 5 mm min^−1^, with rectangular specimens 20 × 8 mm^2^. Joule heating performance was evaluated using a WANPTEK GPS3010D power supply, and the corresponding infrared images were recorded using a Hikmicro HM-TPH21Pro-3AF thermal camera. The complex permittivity and permeability of the composites were measured by waveguide method using an Agilent E5071C vector network analyzer (X-band, 8.2–12.4 GHz). GHz band EMI shielding parameters were obtained on the same instrument over 8.2–12.4 GHz.

Terahertz shielding performance was evaluated using the TP800 time-domain spectrometer system in both reflection and transmission modes. Terahertz waves were focused on a sample with a spot size of 2.5 mm. A Ti: sapphire laser with a central wavelength of 800 nm and a pulse width of 50 fs served as the excitation source. The measurement frequency window spanned 0.2–2.0 THz. The EMI shielding performance was calculated based on the reflectance (*R*), absorbance (*A*), and transmittance (*T*), using the following relationships among *R*, *A*, and *T*:

1 A=1-R-T

*R* and *T* were determined using the THz time-domain spectroscopy (THz-TDS) system in reflection and transmission modes, and the SE_A_ (shielding effectiveness due to absorption), SE_R_ (shielding effectiveness due to reflection), and SE_T_ (total shielding effectiveness) values are calculated as follows:

2 $${\mathrm{SE}}_{T} = - 10\log T$$

3 $${\mathrm{SE}}_{R} = - 10\log (1 - R)$$

4 $${\mathrm{SE}}_{A} = {\mathrm{SE}}_{T} - {\mathrm{SE}}_{R}$$

*Simulation of Radar Cross Section (RCS):* The RCS values were calculated using CST STUDIO SUITE 2023 software. The RCS values (σ) are typically determined by *θ* and φ in the spherical coordinate system, as shown below [25]:

5 $$\sigma \left( {dBm^{2} } \right) = 10\log \left[ {\frac{4\pi S}{{\lambda^{2} }}\left| {\frac{{E_{s} }}{{E_{i} }}} \right|^{2} } \right]$$

where *S*, *λ*, *E*_*s*_, and *E*_*i*_ represent the area of the simulated plate, the wavelength of the incident electromagnetic wave, the scattered field intensity of the transmitted waves, and the incident field intensity of the received waves, respectively.

*Electromagnetic Simulation Method:* Finite element analysis (FEA) of the electromagnetic response was carried out using the frequency domain solver in the RF Module of COMSOL Multiphysics. Two structures of identical volume but distinct morphologies with an isotropic homogeneous configuration and a layered-gradient architecture were modeled under electromagnetic excitation. A 3D geometric model was constructed based on the specified dimensions, and the electromagnetic parameters of the materials were defined over the excitation frequency range (8.2–12.4 GHz). A linearly polarized plane wave was applied as the background excitation source, and periodic boundary conditions were imposed on the lateral surfaces.

The electromagnetic field distribution and characteristics before and after entering the structure are calculated based on Maxwell’s wave equation:

6 $$\nabla \times \mu_{r}^{ - 1} \left( {\nabla \times E} \right) - k_{0}^{2} \left( {\varepsilon_{r} - \frac{j\sigma }{{\omega \varepsilon_{0} }}} \right)E = 0$$

where *E*, *k*_0_, *μ*_*r*_, *ε*_*r*_, *σ*, *ω*, and ε_0_ represent the electric field intensity (V m^−1^), the free space wave number (rad m^−1^), the relative permeability, the relative permittivity, the electrical conductivity (S m^−1^), angular frequency (rad s^−1^), and the Vacuum permittivity (8.854 × 10^–12^ F m^−1^), respectively.

Assuming that the effect of the air medium on the electromagnetic distribution is negligible, $\varepsilon_{r} = \left( {n - {\mathrm{ik}}} \right)^{2}$, σ=0, $\mu_{r} = 1$. were used for the surrounding medium. The geometric model was meshed using a physics-controlled, refined mesh, and steady-state frequency domain calculations were performed to obtain the spatial distributions of the electromagnetic fields and the associated dissipation characteristics.

## Results and Discussion

### Construction of Bioinspired Layered-Gradient Architecture

**Fig. 1:**
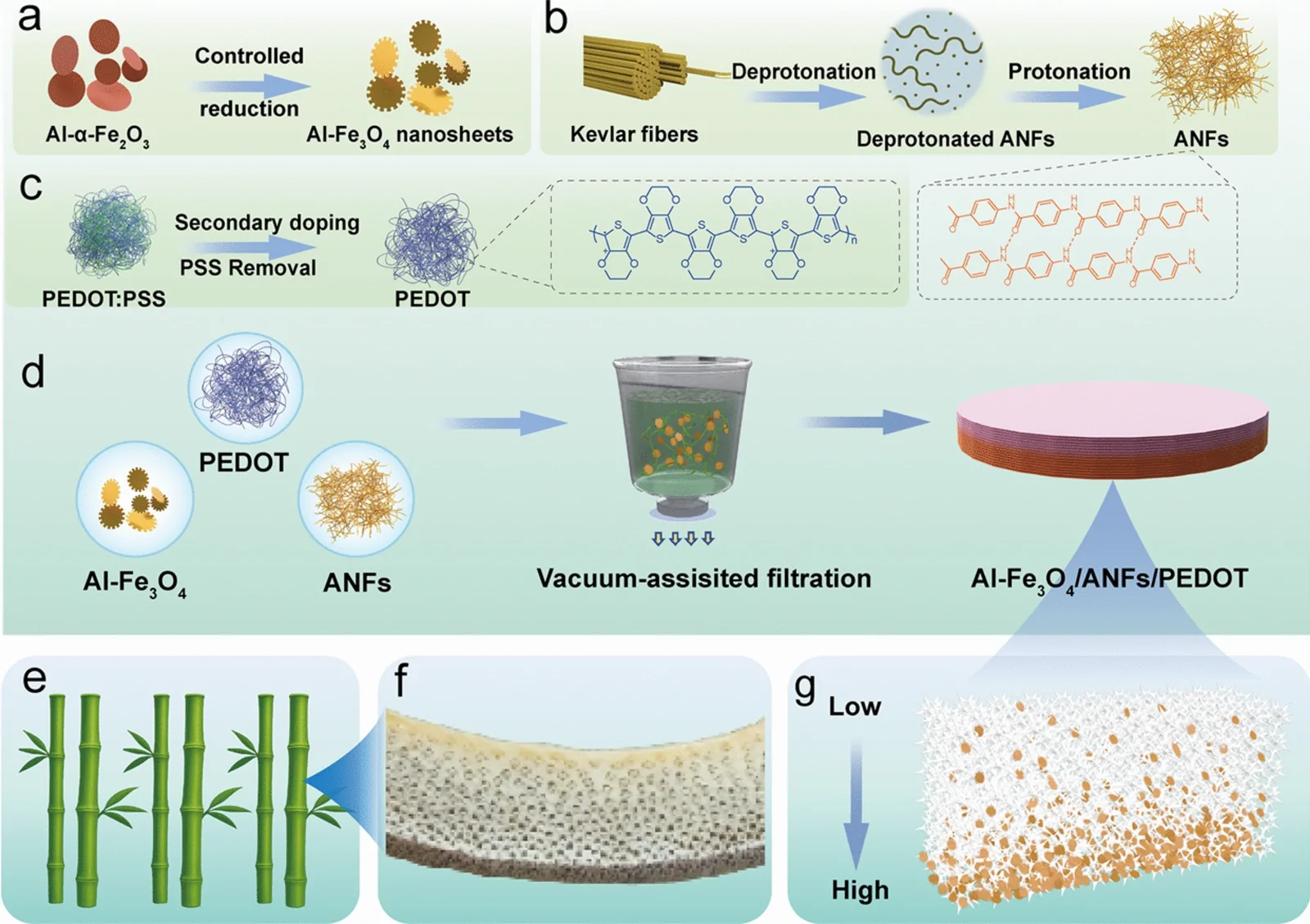
Bioinspired fabrication and gradient architecture of Al-Fe_3_O_4_/ANFs/PEDOT composites. **a** Synthesis of Al-Fe_3_O_4_ nanosheets. **b** Fabrication of ANFs. **c** Schematic illustration of the post-treatment process for PSS removal from PEDOT: PSS. **d** Solution-processed assembly of Al-Fe_3_O_4_/ANFs/PEDOT composite film. **e** Optical images of natural bamboo and** f** its cross section, highlighting the intrinsic hierarchical, radial gradient that informs our materials design. **g** Schematic illustration of the resulting bamboo-inspired, through-thickness gradient structure in the Al-Fe_3_O_4_/ANFs/PEDOT composite film

The construction of the Al-Fe_3_O_4_/ANFs/PEDOT composite with a hierarchically layered architecture is governed by programmable interfacial chemistry and mesoscale assembly among its constituents. As schematically illustrated in Fig. 1b, the deprotonation of aramid microfibers disrupts the intrinsic hydrogen bonding network, affording a stable, homogeneous colloidal dispersion of individualized ANFs. Subsequent exposure to a polar medium re-establishes hydrogen bonding, driving their controlled reassembly into high aspect ratio ANFs. Owing to their excellent processability, mechanical robustness, and abundant surface functionalities, ANFs serve as a versatile polymeric scaffold for engineering high-performance composites [27, 28]. To impart electrical conductivity, PEDOT:PSS is employed as an intrinsically conductive polymer. While the PSS component improves aqueous dispersibility, its excess severely compromises electrical conductivity. A secondary doping and PSS decoupling treatment using dodecylbenzene sulfonic acid (DBSA) and dimethyl sulfoxide (DMSO) selectively extracts PSS and promotes conformational rearrangement of PEDOT chains, thereby markedly enhancing charge transport (Fig. 1c). In parallel, ultrathin magnetic Al-*α*-Fe_2_O_3_ nanosheets are synthesized via a hydrothermal process, wherein selective adsorption of Al^3+^ biases anisotropic growth toward a two-dimensional morphology. A subsequent controlled reduction step converts these into Al-Fe_3_O_4_ nanosheets featuring a distinctive sawtooth-like edge topology (Fig. 1a). The substitution of Fe^3+^ by Al^3+^ induces lattice contraction and electronic structure modulation, which has been systematically demonstrated in our previous work [25]. In brief, analyses confirm that Al^3+^ is successfully incorporated into the Fe_3_O_4_ lattice, leading to reduced interplanar spacing, stable valence states, and suppressed saturation magnetization. These structural and magnetic variations collectively give rise to redistributed charge density and perturbed magnetic exchange interactions, thereby enhancing dielectric polarization, tuning magnetic response, and ultimately enabling improved electromagnetic attenuation capability. The sawtooth edges further enhance localized field concentration and interfacial polarization. These components are uniformly co-dispersed by high-shear blending and assembled into free-standing films via vacuum-assisted filtration, yielding a hierarchically stratified through-thickness gradient architecture (Fig. 1d). This bottom-up fabrication route is modular and scalable, establishing a general platform for fabricating anisotropic multifunctional composites.

Drawing inspiration from biological archetypes, the resultant Al-Fe_3_O_4_/ANFs/PEDOT films emulate the radially gradient architecture of bamboo (Fig. 1e). In its natural state, bamboo concentrates load-bearing cellulose fibers near the outer wall within a lignin–hemicellulose matrix (Fig. 1f), thereby optimizing stiffness and fracture toughness. Analogously, the filtration-driven assembly creates a monotonic decrease in the concentrations of Al-Fe_3_O_4_ nanosheets and PEDOT from the filtration-inlet side (bottom) to the opposite surface (top), forming a precise, bamboo-like compositional gradient (Fig. 1g). Al-Fe_3_O_4_ nanosheets, owing to their higher density, preferentially migrate toward the filter membrane and become enriched at the bottom, while ANFs with a high aspect ratio concurrently construct a continuous network scaffold. The components undergo a dynamic process of migration, retention, and redistribution under the influence of intermolecular interactions and fluid shear forces, ultimately forming a stable asymmetric distribution along the thickness direction. The gradient variation rate is governed by both intrinsic material properties and processing parameters. Intrinsic factors include the density, size, morphology, surface charge states, and interfacial interactions such as hydrogen bonding and electrostatic interactions of the components. Processing parameters include vacuum pressure, filtration rate, filtration time, and dispersion concentration. By rationally tuning these parameters, the gradient architecture and its variation tendency can be controllably regulated. The emergent impedance gradient and anisotropic filler distribution confer directionally dependent functionalities. The compositionally enriched bottom face facilitates enhanced charge transport and magnetic-dielectric coupling, enabling efficient EMW attenuation, whereas the asymmetric cross section introduces an intrinsic bias for stimulus-responsive actuation. Such deliberate spatial programming is pivotal for integrating low areal density, mechanical flexibility, and anisotropic responsiveness within a unified material system.

**Fig. 2:**
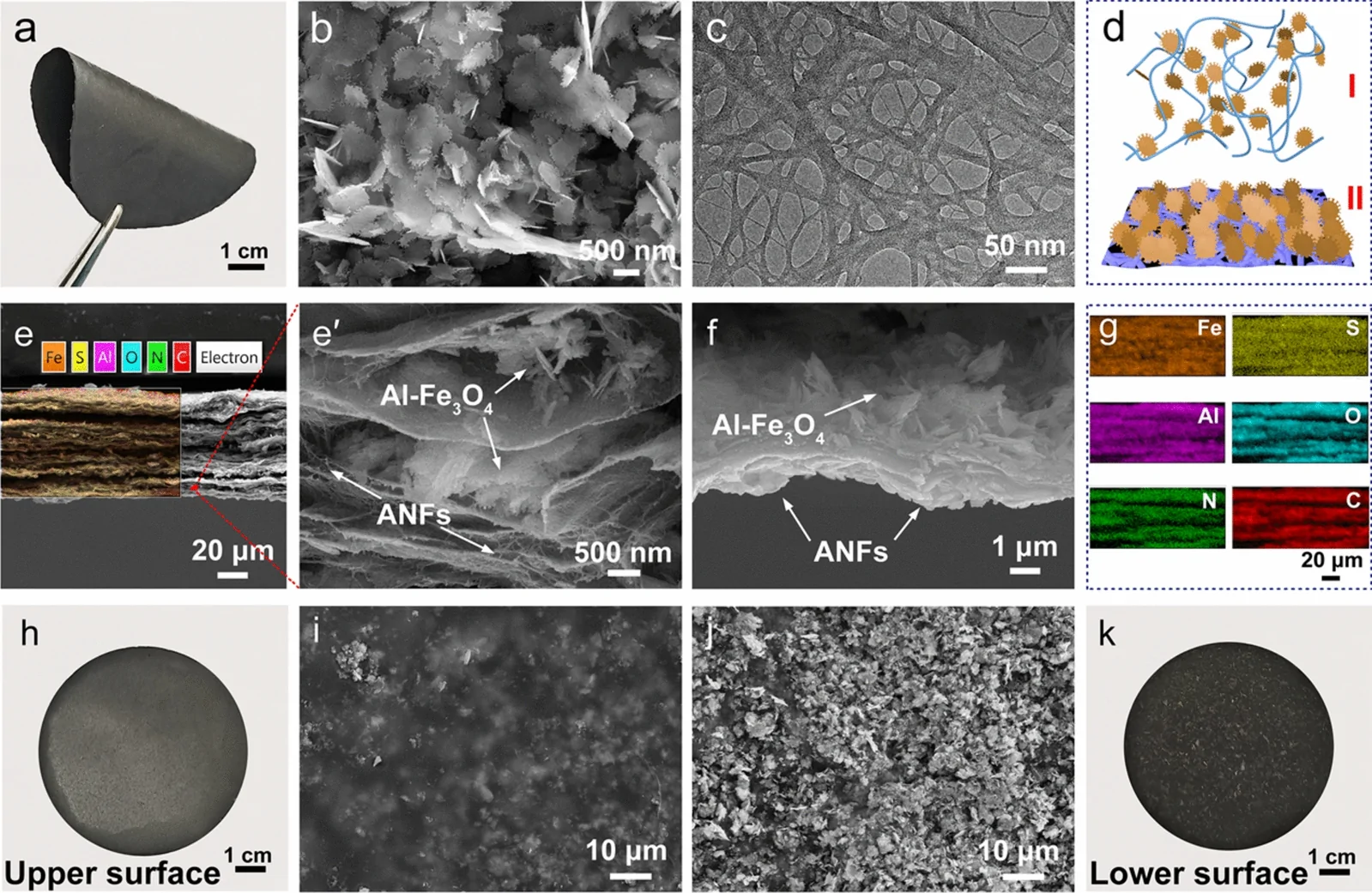
Morphology, gradient architecture, and surface anisotropy of bamboo-inspired Al-Fe_3_O_4_/ANFs/PEDOT composite film. **a** Optical photograph of a free-standing, conformally curved Al-Fe_3_O_4_/ANFs/PEDOT composite film **b** SEM image revealing the characteristic sawtooth-edged morphology of Al-Fe_3_O_4_ nanosheets. **c** TEM image of ANFs. **d** Schematic illustrating two representative through-thickness configurations in the layered-gradient Al-Fe_3_O_4_/ANFs/PEDOT composite film. **e** Cross-sectional SEM image and corresponding elemental mapping image of the gradient composite. **e′** magnified view highlighting the through-thickness stratification. **f** Higher-magnification cross-sectional SEM image of the inner region of the bottom layer. **g** Elemental mapping image confirming the spatial distribution of the constituent elements across the section. **h, k** Optical images of the top (upper) and bottom (lower) surfaces, respectively. **i, j** Corresponding SEM images of the top and bottom surfaces, underscoring the pronounced contrast in surface textures imparted by the bamboo-inspired gradient design

The microstructure and physical attributes of the composite are systematically illustrated in Fig. 2. An optical photograph (Fig. 2a) underscores the film’s high flexibility, a critical property for conformal integration with deformable devices. High-resolution imaging of the Al-Fe_3_O_4_ nanosheets (Fig. 2b) reveals well-defined sawtooth edges with high morphological uniformity and nanoscale sharpness across the entire sample. Al doping, in concert with the edge geometry, serves as an effective lever to tune to precisely tune the material’s electromagnetic parameters, thereby boosting EMW attenuation and energy dissipation efficiency. Simultaneously, the ANFs reconstruct into a percolated, three-dimensional nanofibrillar network (Fig. 2c), extending the utility of aramid beyond traditional fiber applications. Critically, the synergistic coupling between the 3D ANFs scaffold and the sawtooth-edged Al-Fe_3_O_4_ nanosheets mediated by hydrogen bonding, van der Waals interactions, and mechanical interlocking establishes a robust confinement effect. This effect immobilizes fillers, suppresses aggregation, and stabilizes the spatial distribution. The resulting interfacial heterogeneity intensifies interfacial polarization and promotes multiple internal scattering/reflection under EMW irradiation, collectively enhancing electromagnetic energy dissipation. Cross-sectional SEM analysis of the Al-Fe_3_O_4_/ANFs/PEDOT film (Fig. 2e) reveals a sharply delineated, hierarchically stratified architecture templated by the ANFs framework.

Within this structure, two characteristic embedding motifs for the Al-Fe_3_O_4_ nanosheets are discernible (Fig. 2e′, f). At higher magnification, the serrated sawtooth edge topology of the nanosheets is clearly resolved, underscoring their distinctive morphology and high interfacial activity. In the first motif (Fig. 2e; schematic in Fig. 2d(I)), Al-Fe_3_O_4_ nanosheets are uniformly distributed within the porous, percolating ANFs network. This open architecture stabilizes dispersion, maximizes accessible magnetic-dielectric interfacial area, and facilitates efficient modulation of the complex electromagnetic parameters, thereby enriching attenuation pathways. In the second motif, prevalent within the interior of the bottom stratum (Fig. 2f, d(II)), strong intermolecular hydrogen bonding compacts the ANFs into a dense lamella that tightly immobilizes the nanosheets. This closely packed assembly provides mechanical reinforcement while simultaneously intensifying interfacial polarization and refining impedance matching. The coexistence of these two interaction modes open-network anchoring and dense-layer confinement endows the composite with synergistic functionality. Structural confinement suppresses filler migration and aggregation, while interfacial engineering amplifies Maxwell–Wagner–Sillars polarization and multiple scattering. Collectively, these effects underpin the film’s superior electromagnetic response, mechanical stability, and stimulus-responsive behavior.

**Fig. 3:**
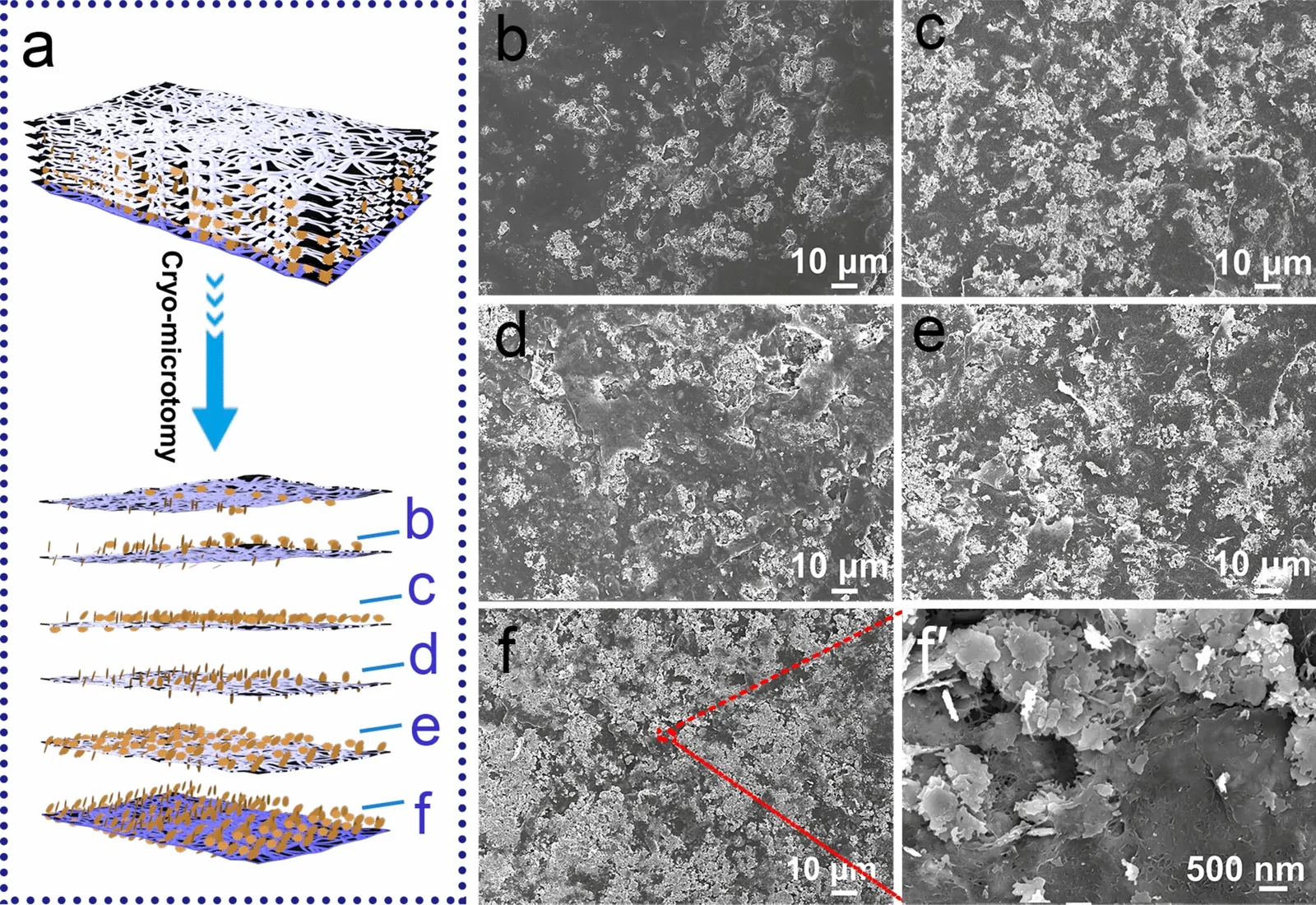
Verification of the through-thickness gradient architecture by cryo-microtomy. **a** Schematic illustration of the cryo-microtomy procedure applied to the Al-Fe_3_O_4_/ANFs/PEDOT composite film. SEM images of the film sequentially sectioned into five intermediate layers:** b** first layer, **c** second layer, **d** third layer, **e** fourth layer, and **f** fifth layer, with **f′** the corresponding magnified view

Elemental mapping across the Al-Fe_3_O_4_/ANFs/PEDOT film thickness provides direct evidences a well-orchestrated through-thickness gradient (Fig. 2e, g). However, the absence of a pronounced stepwise gradient in the EDS signal is primarily attributed to the resolution limitations of the technique and the inherently continuous nature of the gradient, where composition varies gradually across the thickness. Macroscopic images of the two faces (Fig. 2h, k) display pronounced visual contrast, which is corroborated by SEM analysis of the corresponding surfaces (Fig. 2i, j). The bottom face is markedly enriched in Al-Fe_3_O_4_ nanosheets compared to the top face. High-magnification SEM images (Fig. S1a, b) confirm a monotonic decline in nanosheet areal density from bottom to top, characteristic of the bamboo-inspired architecture. Low-magnification SEM images (Fig. S1c, d) further illustrate the overall distribution trend on a larger scale. To overcome the limitations of localized SEM observation and provide a more comprehensive view of the gradient architecture, cryo-microtomy analysis was performed on the composite film, which further reveals an increasing trend of Al-Fe_3_O_4_ nanosheets from the top to the bottom layers (Fig. 3). Quantitative evaluation (Fig. S2) of the nanosheet area fraction based on the cryo-microtomy SEM images unequivocally confirms the successful construction of the gradient structure. Although present at modest levels, PEDOT likewise exhibits a through-thickness gradient and acts cooperatively with the magnetic framework. At low loadings, PEDOT improves impedance matching and strengthens dielectric loss; with increasing content, the elevated conductivity substantially enhances EMI shielding effectiveness. These observations highlight a cooperative triad Al-Fe_3_O_4_ nanosheets, ANFs scaffold, and gradient PEDOT that yields a structurally integrated, multifunctional composite with superior electromagnetic performance.

### Gradient-Induced Anisotropic Actuation Behavior

**Fig. 4:**
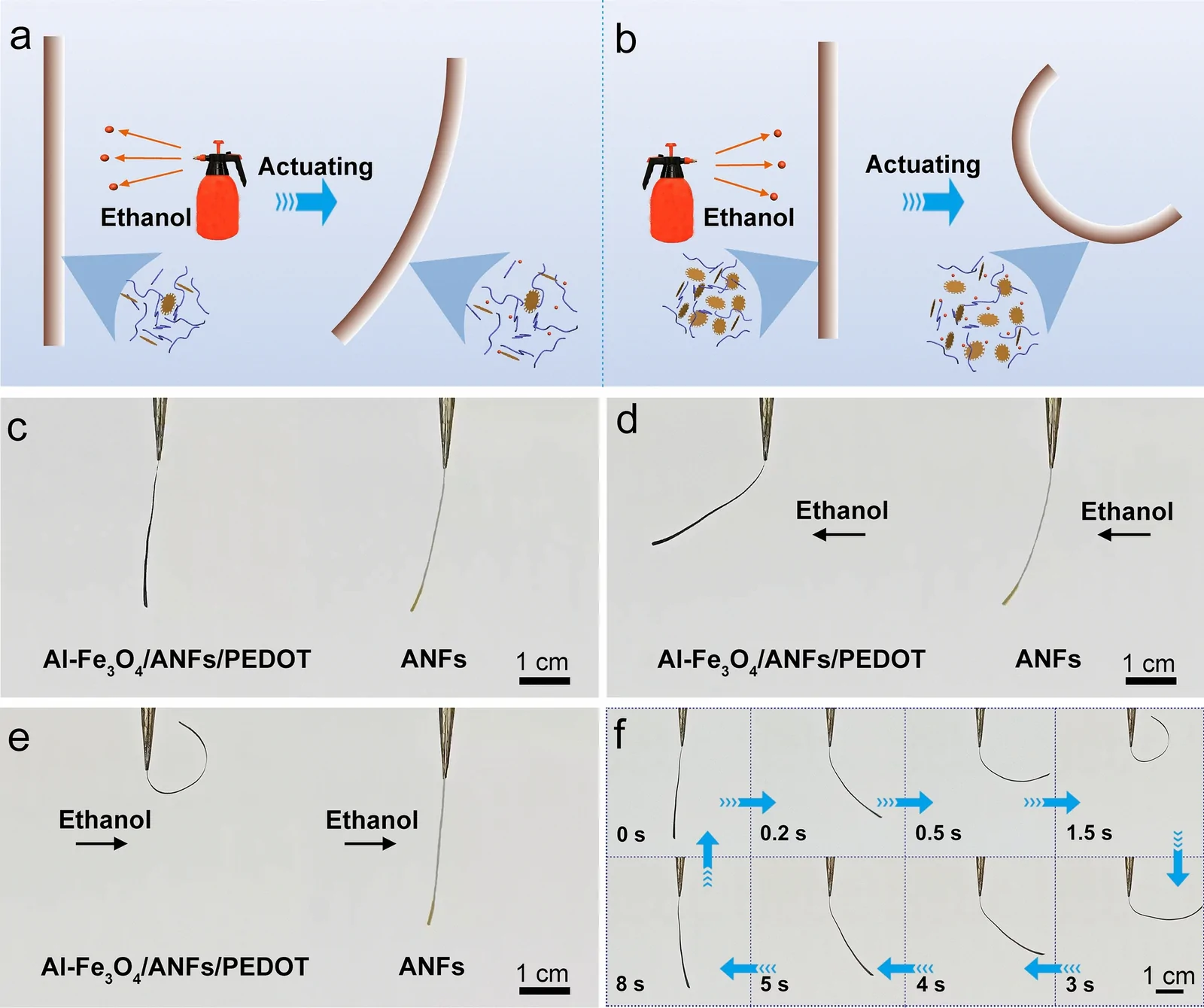
Face-selective, ethanol-triggered actuation of Al-Fe_3_O_4_/ANFs/PEDOT composite film. Schematics of interfacial ethanol uptake and differential swelling at the **a** top (upper) and **b** bottom (lower) surfaces, elucidating the asymmetric actuation mechanism. **c** Optical image of the free-standing film in its as-prepared state. Time-resolved optical images showing ethanol-responsive bending behavior of the **d** top and **e** bottom faces for both the Al-Fe_3_O_4_/ANFs/PEDOT and ANFs. **f** Representative actuation cycle recorded from the bottom face under repeated ethanol exposure, highlighting rapid, reversible bending-recovery behavior and robust cyclic stability

Beyond electromagnetic regulation, the gradient morphology imparts distinctive, direction-selective actuation capabilities. Upon exposure to ethanol vapor, the films undergo rapid, reversible bending (Fig. 4a, b), driven by solvent uptake within the ANFs matrix and subsequent differential swelling/shrinkage during absorption–evaporation cycles (Movie S1). The intrinsic compositional and morphological asymmetry between the two faces (Fig. 2i, j) gives rise to unequal sorption kinetics and strain development across the thickness. The Al-Fe_3_O_4_-rich bottom surface, characterized by higher roughness and defect density, permits faster ethanol ingress and greater volumetric expansion [29]. In contrast, the top surface, dominated by densely packed ANFs, exhibits reduced porosity and imposes a barrier to solvent diffusion. The lower interfacial energy and higher diffusion coefficient of ethanol in ANFs compared to PEDOT lead to preferential swelling of the ANFs-rich layer, while the PEDOT content in the actuation-testing Al-Fe_3_O_4_/ANFs/PEDOT sample is too low (only 0.004 mL) to significantly affect the overall swelling behavior. Therefore, this gradient in swelling strain drives directional bending result in the observed ethanol-triggered anisotropic actuation. As shown in Fig. 4c, the free-standing film in its initial, flat state exhibits a uniform and intact appearance, highlighting the structural integrity of the layered-gradient architecture prior to stimulus application. The resulting strain gradient generates pronounced curvature (Fig. 4d, e). Quantitative analysis of the actuation dynamics reveals exceptional responsiveness. The bending angle increases from 58° at 0.2 s to 111° at 0.5 s, reaching 238° at 1.5 s, corresponding to an average angular velocity of ~ 158 s^−1^ during the rapid actuation stage (0–1.5 s). A complete expansion–contraction cycle concludes within 8 s due to rapid ethanol diffusion and evaporation (Fig. 4f), with an average recovery speed of ~ 37° s^−1^. In contrast, the top surface (dense ANFs layer) exhibits a markedly reduced actuation response, with a maximum bending angle of only ~ 55° at 1.5 s and a significantly lower average angular velocity of ~ 37° s^−1^, highlighting the strong anisotropy arising from the through-thickness gradient structure. The detailed quantitative actuation performance is summarized in Table S1. The actuator retains mechanical integrity and actuation amplitude over repeated cycles, demonstrating excellent fatigue resistance. Therefore, the same gradient, impedance-engineered architecture that optimizes EMW management performance simultaneously encodes solvent-responsive, anisotropic actuation, positioning Al/Fe_3_O_4_/ANFs/PEDOT films as compelling candidates for multifunctional soft electronics and electromagnetic skin applications.

### Mechanical, Thermal, and Joule Heating Properties

The functional versatility of the Al-Fe_3_O_4_/ANFs/PEDOT films extends beyond solvent-triggered actuation to encompass a spectrum of multifunctionalities, including mechanical robustness, thermal stability, Joule heating, and superior EMW absorption. Tensile stress–strain curves (Fig. S3a) indicate an average tensile strength of 33.9 MPa with an elongation at break of 9.7%, striking a remarkable balance between strength and ductility. Systematic variation of PEDOT content (Fig. S3b) initially reduces both strength and elongation, attributable to perturbation of the percolated ANFs network. A partial recovery is observed at higher loadings as PEDOT establishes conductive bridging pathways, highlighting the intricate interplay between structural integrity and electrical percolation. The films demonstrate notable load-bearing capacity, supporting a 1000 g mass without structural failure (Fig. S4). Thermogravimetric analysis (Fig. S5) shows that ceramic Al-Fe_3_O_4_ nanosheets elevate the onset of thermal decomposition and suppress volatile evolution [30]. Meanwhile, increased PEDOT content raises the char yield at 800 °C, consistent with enhanced carbonaceous residue formation and improved thermal endurance.

The integration of PEDOT also confers outstanding electrothermal performance. The equilibrium temperature during Joule heating increases approximately linearly with applied voltage. The Al-Fe_3_O_4_/ANFs/PEDOT_2_ film rapidly reaches 233 °C at 20 V (Fig. S6), underscoring its potential for compact, high-power-density thermal management. Notably, the bamboo-like gradient architecture remains intact in both Al-Fe_3_O_4_/ANFs/PEDOT_2_ (Fig. S7) and Al-Fe_3_O_4_/ANFs/PEDOT_3_ (Fig. S8), suggesting that mesoscale anisotropy contributes not only to efficient heat generation but also to directional heat dissipation. To decouple the intrinsic architectural features from drying-induced artifacts, supercritical drying was employed to eliminate capillary stresses, thereby preserving the native porous morphology. The resulting aerogel structure (Fig. S9) reveals a well-maintained 3D ANFs scaffold conformally coated by a thin PEDOT. Compared to the uncoated aerogel (Fig. S9a, a′), the average fiber diameter increases from 20 to 42 nm, confirming a core–shell nanofibrillar configuration. Such conformal coating ensures intimate interfacial contact between PEDOT and ANFs, which is further reinforced by synergistic non covalent interactions (e.g., hydrogen bonding, π-π stacking, and electrostatic attraction) [31]. This robust interfacial coupling not only facilitates the construction of an interconnected, low resistance percolating network that homogenizes current distribution and thermal flux, enabling rapid, stable, and spatially uniform Joule heating, but also introduces abundant heterogeneous interfaces that contribute to enhanced interfacial polarization. Consequently, the coupling of a porous 3D framework with nanoscale conductive coatings provides a synergistic strategy that optimizes charge transport, dielectric loss, and electrothermal conversion.

### Microwave Absorption Performance and Mechanism

**Fig. 5:**
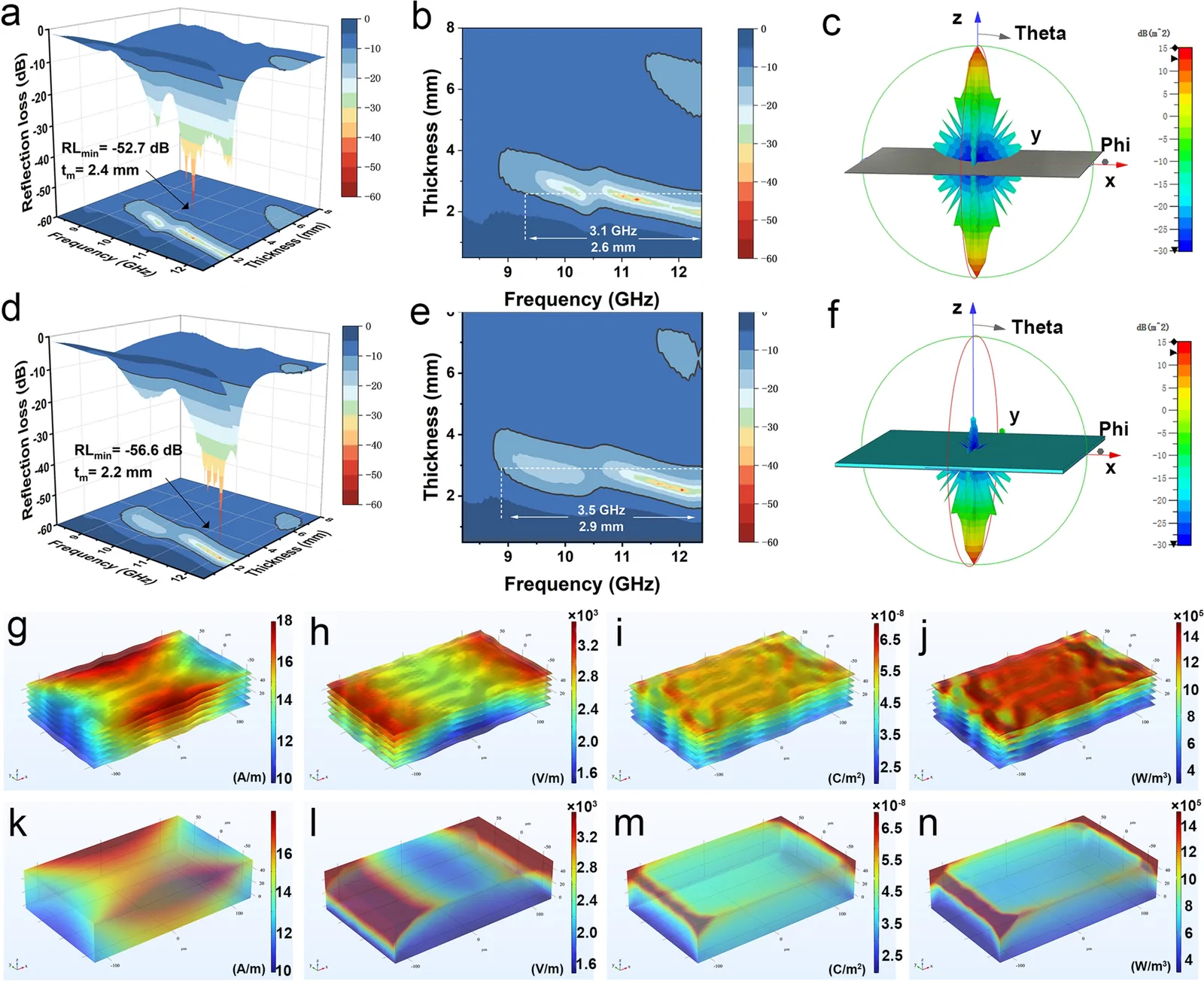
Broadband electromagnetic attenuation and stealth performance enabled by layered-gradient architecture. **a** Three-dimensional reflection loss curves maps and **b** two-dimensional projection diagrams (RL) of Al-Fe_3_O_4_/ANFs composites. **c** Three-dimensional radar cross-section (RCS) plot of a perfect electric conductor (PEC) reference. **d** Three-dimensional reflection loss curves maps and **e** corresponding two-dimensional RL of Al-Fe_3_O_4_/ANFs/PEDOT composites. **f** PEC coated with an Al-Fe_3_O_4_/ANFs/PEDOT absorber, evidencing pronounced backscatter suppression. **g-j** Simulated spatial distributions of magnetic field, electric field, polarization loss, and conduction loss power within the layered-gradient Al-Fe_3_O_4_/ANFs/PEDOT architecture; **k-n** the corresponding maps for an isotropic counterpart, underscoring the gradient-induced enhancement of energy dissipation pathways

A cornerstone of this work is the exceptional EMW absorption performance of Al-Fe_3_O_4_/ANFs/PEDOT films, a property governed by their hierarchical gradient structure and synergistic loss mechanisms. According to transmission line theory [32], the reflection loss (RL) can be calculated using the following equations:

7 $$Z_{{{\mathrm{in}}}} = Z_{0} \sqrt {\frac{{\mu_{r} }}{{\varepsilon_{r} }}} \tanh \left[ {j\left( {\frac{2\pi fd}{c}} \right)\sqrt {\mu_{r} \varepsilon_{r} } } \right]$$

8 $${\mathrm{RL}} = 20\log \left| {\left( {Z_{{{\mathrm{in}}}} - Z_{0} } \right)/\left( {Z_{{{\mathrm{in}}}} + Z_{0} } \right)} \right|$$

where *Z*_in_ represents the normalized input impedance, *Z*_*0*_ is the free space impedance, *ε*_*r*_ and *μ*_*r*_ are the relative complex permittivity and permeability, respectively, *f* is the microwave frequency, *d* is the absorber thickness, and *c* is the speed of light. Key performance metrics include the minimum reflection loss (RL_min_) and the effective absorption bandwidth (EAB < −10 dB) [33–35]. The Al-Fe_3_O_4_/ANFs baseline achieves an RL_min_ of − 52.7 dB at 2.4 mm and an EAB of 3.1 GHz at 2.6 mm (Fig. 5a, b). Incorporation of PEDOT further enhances performance, with the Al-Fe_3_O_4_/ANFs/PEDOT composite reaching an RL_min_ of − 56.6 dB at 2.2 mm and an EAB of 3.5 GHz at 2.9 mm (Fig. 5d, e), as summarized in Table S1. Two-dimensional (2D) RL contour maps (Fig. S10) corroborate these improvements, which originate from elevated dielectric and conductive losses imparted by PEDOT. To further validate the underlying mechanisms, the experimental impedance matching characteristics are presented in Fig. S10c, showing good agreement with the simulated electromagnetic field attenuation behavior. The optimized impedance matching enables efficient penetration of incident waves, consistent with the simulated progressive attenuation observed within the gradient architecture, thereby supporting the synergistic role of impedance regulation and multiscale energy dissipation. The magnetic hysteresis loops of the composite films are presented in Fig. S11. As shown in Fig. S12, the magnetic loss factor (tan δ_μ_) consistently exceeds the dielectric loss factor (tan δ_ε_), indicating magnetically dominated dissipation [36]. Nevertheless, excessive PEDOT leads to over-percolation and severe impedance mismatch, driving RL toward 0 dB in Al-Fe_3_O_4_/ANFs/PEDOT_2_ and Al-Fe_3_O_4_/ANFs/PEDOT_3_ (Fig. S13a, b). Accordingly, a low PEDOT loading of 0.004 mL was adopted for the absorption-oriented film to maintain moderate conductivity and optimal impedance matching. Therefore, precise compositional optimization is essential to preserve impedance matching while sustaining multi-channel loss pathways [37, 38].

To evaluate application-relevant stealth characteristics, radar cross-section (RCS) simulations were performed on a perfect electric conductor (PEC) plate coated with 2.5 mm thick Al-Fe_3_O_4_/ANFs/PEDOT layer under normal incidence along the negative z-axis. In the adopted coordinate system, the x-axis and z-axis correspond to phi = 0° and theta = 0°, respectively, with EMW incidence aligned along the negative z-axis direction. At 11.5 GHz, the three-dimensional (3D) scattering patterns (Fig. 5c, f) display a pronounced suppression of scattered energy for the coated PEC relative to bare substrate, evidencing substantially enhanced EMW absorption. The corresponding 2D RCS curves (Fig. S14a) show a reduction from 12.7 dB m^2^ (bare PEC) to − 18.2 dB m^2^ for the coated system at normal incidence. Angle-dependent RCS attenuation values at 0°, 20°, 40°, and 60° are 30.9, 47.8, 29.9, and 6.7 dB m^2^ respectively (Fig. S14b), highlighting broadband, angle-tolerant stealth performance. Collectively, these results confirm that the gradient-structured film effectively suppresses both specular and diffuse scattering.

To further elucidate the role of the layered gradient, finite-element simulations at 10 GHz were conducted to visualize field distributions and energy dissipation. This frequency is selected as it lies near the center of the X-band (8.2–12.4 GHz), thereby minimizing boundary-frequency effects and enabling a more representative evaluation of the intrinsic electromagnetic response. Moreover, according to the reflection loss results, the material exhibits strong attenuation within this frequency region, making it particularly suitable for capturing dominant energy dissipation behaviors. Two idealized absorbers were considered: an anisotropic model representing the Al-Fe_3_O_4_/ANFs/PEDOT structure, and an isotropic control. Both were subjected to normal incidence at the top surface. The layered-gradient architecture exhibits progressive attenuation of both magnetic and electric field intensities across the through-thickness direction (Fig. 5g, h), whereas the isotropic control exhibits edge-localized fields and limited penetration (Fig. 5k, l). Enhanced polarization under the induced electric field in the layered-gradient film (Fig. 5i, m) translates into stronger conduction loss (Fig. 5j), while the impedance-mismatched isotropic sample admits less EMW ingress and therefore exhibits weaker resistive dissipation (Fig. 5n).

The superior absorption stems from hierarchical impedance modulation coupled with multiscale loss pathways. The gradient architecture establishes a continuous variation in complex permittivity and electrical conductivity along the thickness direction, enabling progressive impedance matching from the air-incident interface to the interior. A filler-lean entrance surface approaches optimal impedance matching with air, enabling efficient coupling of incident waves into the material. As EMW propagate into filler-richer strata, multiple scattering, Maxwell-Wagner-Sillars interfacial polarization, and internal reflections are progressively intensified, lengthening the propagation path and amplifying energy dissipation. Abundant heterogeneous interfaces among Al-Fe_3_O_4_, ANFs, and PEDOT further promote interfacial polarization under alternating fields, while magnetic nanosheets contribute eddy-current losses and high-frequency magnetic resonances (natural and exchange) [39]. In summary, the combination of microscopic loss mechanisms with macroscopic gradient engineering yields exceptional attenuation, establishing the Al-Fe_3_O_4_/ANFs/PEDOT system as a high-performance EMW absorber for advanced stealth and EMI shielding applications.

To contextualize the EMW absorption performance of the Al-Fe_3_O_4_/ANFs/PEDOT composite, a benchmark comparison with representative absorbers reported in the literature is summarized in Table 1. Conventional powders, aerogels, and aerogel-filled films often sacrifice flexibility and mechanical integrity to achieve strong attenuation, and rarely realize genuine multifunctional integration. By contrast, the present flexible film combines a very low RL_min_, broad effective absorption bandwidth (EAB), and a practical thickness, surpassing several state-of-the-art systems while retaining structural compliance and processability. This well-balanced combination of mechanical accommodation and electromagnetic dissipation underscores the promise of the Al-Fe_3_O_4_/ANFs/PEDOT for next-generation flexible EMW-absorbing materials.

**Table 1:** X-band (8–12 GHz) EMW absorption metrics of the Al-Fe_3_O_4_/ANFs/PEDOT composite benchmarked against representative state-of-the-art absorbers reported in the literature

Absorbers	Structure	Minimum reflection loss		Effective absorption bandwidth		Reference
		RL_min_ (dB)	Thickness (mm)	EAB (GHz)	Thickness (mm)	
MXene/ANF/PI	Aerogel	− 39.6	2.4	3.1	2.6	[40]
CF@NiCo_2_O_4_@MnO_2_	Aerogel	− 27.8	2.7	4.2	3.2	[41]
rGO/FeCo-LDH/PPy	Aerogel	− 45.0	3.5	4.2	3.5	[42]
ZnO/SiCnw	Aerogel	− 42.4	4.0	4.2	4.0	[43]
CFA@H-C/Co_3_O_4_	Aerogel	− 43.5	3.0	7.84	3.0	[44]
Ti_3_C_2_T_x_ /cellulose	Hydrogel	− 28.0	2.0	4.0	2.0	[45]
rGO/SiC NW	Foam	− 19.6	3.0	4.2	-	[46]
PF/GN/Fe_3_O_4_	Foam	− 35.0	5.0	4.2	4.5	[47]
copper mesh@Cu_2-x_S@PI	Film	− 25.0	1.6	1.2	1.4	[48]
CoFe_2_O_4_	Powder	− 39.8	2.0	1.0	2.0	[49]
Al-Fe_3_O_4_/ANFs/PEDOT	Flexible film	− 56.6	2.2	3.5	2.2	***This work***

### Broadband EMI Shielding Performance and Mechanism (GHz-THz)

**Fig. 6:**
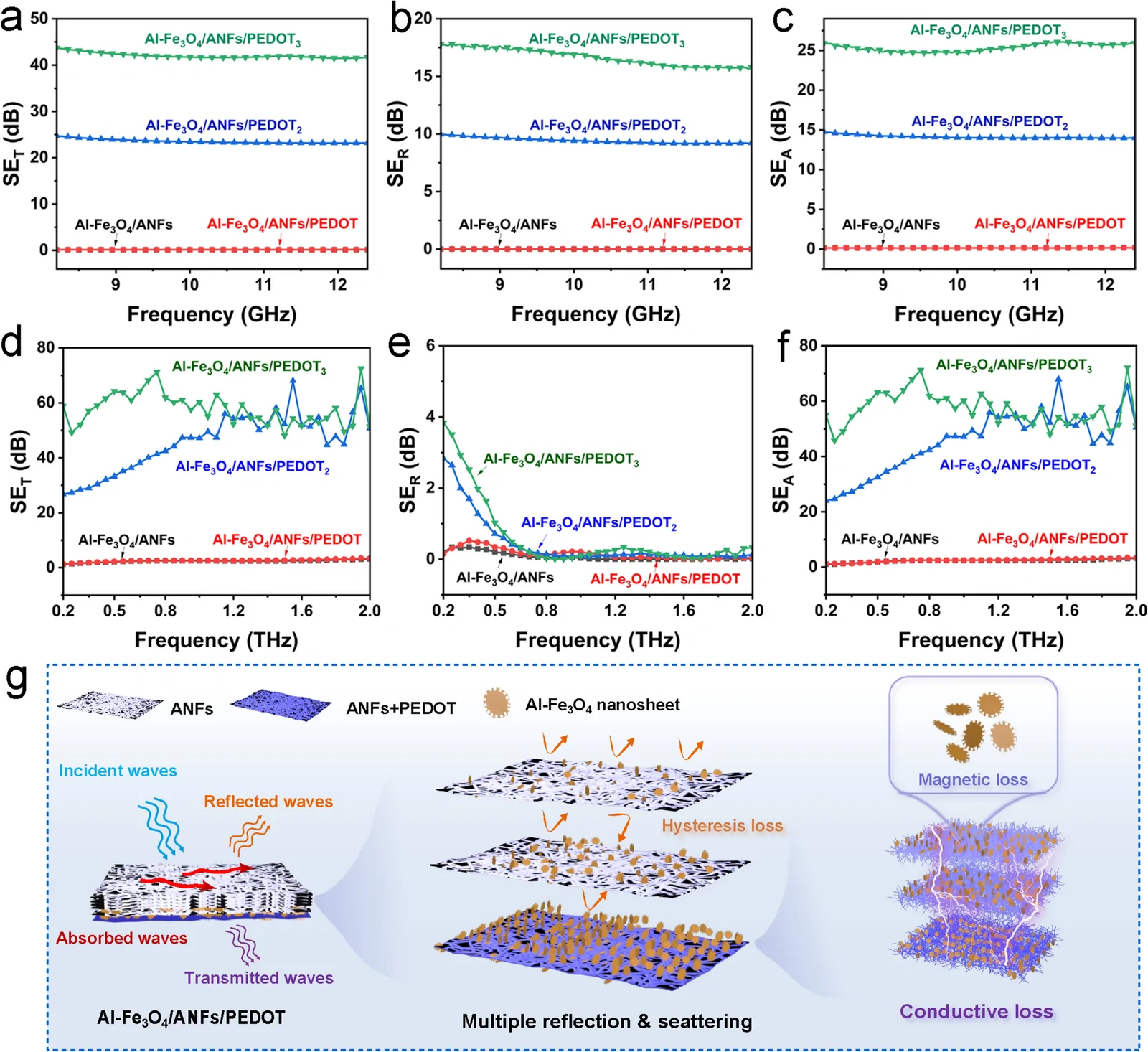
Broadband EMI shielding across GHz and THz regimes and the associated dissipation pathways. **a-c** Frequency-dependent total (EMI SE_T_), reflection (EMI SE_R_), and absorption (EMI SE_A_) shielding effectiveness of the composite films in the GHz band. **d-f** Corresponding SE_T_, SE_R_, and SE_A_ in the THz regime, demonstrating sustained attenuation and stealth-relevant suppression of transmitted power. **g** Schematic illustration of the shielding mechanism in the Al-Fe_3_O_4_/ANFs/PEDOT_3_ laminate: through-thickness impedance matching, hierarchical conductive networks, interfacial Maxwell–Wagner polarization, magnetic (natural and eddy-current) losses, and multiple internal reflections collectively yield absorption-dominant shielding with minimal back-reflection

Building on their strong EMW absorption characteristics, the Al-Fe_3_O_4_/ANFs/PEDOT composites demonstrate equally outstanding EMI shielding across both the GHz and THz regimes. As PEDOT loading increases, the bulk conductivity evolves from insulating (5.89478 × 10^–11^ S m^−1^ for Al-Fe_3_O_4_/ANFs) to highly conductive (975.721 S m^−1^ for Al-Fe_3_O_4_/ANFs/PEDOT_3_), driven by the formation of a continuous PEDOT percolation network (Fig. S15). In the gradient films, the conductivity varies along the thickness direction: the side with lower PEDOT content exhibits lower conductivity due to an incomplete conductive network, while the side with higher PEDOT content forms a continuous percolation pathway, resulting in significantly higher conductivity. This measured value reflects the macroscopic average electrical performance of the entire film, integrating the contributions from each layer along the thickness direction. The corresponding GHz-range shielding components including total shielding (SE_T_), reflection (SE_R_), and absorption (SE_A_) are summarized in Fig. 6a-c. Despite comparable thicknesses (~ 56–83 μm), only composites that exceed the electrical percolation threshold (Al-Fe_3_O_4_/ANFs/PEDOT_2_, and Al-Fe_3_O_4_/ANFs/PEDOT_3_) exhibit pronounced EMI SE, with SE_T_ values 23.5 and 42.0 dB, respectively. Low-conductivity counterparts (Al-Fe_3_O_4_/ANFs, Al-Fe_3_O_4_/ANFs/PEDOT_3_) are approximately 0.1. These trends highlight the critical role of a well-connected conductive network for efficient EMI shielding, while the bamboo-like layered gradient simultaneously preserves mechanical flexibility and enables integrated multifunctionality.

Within the GHz band, the Al-Fe_3_O_4_/ANFs/PEDOT_2_ and Al-Fe_3_O_4_/ANFs/PEDOT_3_ films exhibit EMI SE_R_ values of 9.4 and 16.7 dB, respectively, with corresponding SE_A_ values of 14.1 and 25.4 dB. The predominance of SE_A_ and SE_T_ indicates an absorption-dominated shielding mechanism, a desirable trait for minimizing secondary electromagnetic pollution. The conductivity-driven increase in both SE_A_ and total shielding SE_T_ confirms that PEDOT not only establishes long-range charge transport but also substantially enhances dielectric and conductive losses under alternating electromagnetic fields. Remarkably, the Al-Fe_3_O_4_/ANFs/PEDOT_3_ achieves a high specific shielding efficiency (SE_T_/effective thickness) of approximately 506 dB mm^−1^, surpassing many reported flexible shields and highlighting its suitability for ultrathin, lightweight applications where stringent mass and volume constraints exist.

Beyond the GHz regime, the composites retain strong electromagnetic stealth in the THz band (Fig. 6d-f). Increasing the PEDOT content in Al-Fe_3_O_4_/ANFs/PEDOT_3_ to 1.5 times that in Al-Fe_3_O_4_/ANFs/PEDOT_2_ elevates the average SE_T_ from 45.8 to 57.8 dB. This enhancement is attributed to the formation of denser conductive pathways and an increased density of interfacial dipolar sites, which collectively intensify dielectric polarization and energy dissipation. Consistent with the GHz band behavior, THz shielding remains absorption-dominant. The EMI SE_R_ values for the Al-Fe_3_O_4_/ANFs/PEDOT_2_ and Al-Fe_3_O_4_/ANFs/PEDOT_3_ composites are minimal (0.5 and 0.6 dB, respectively), while SE_A_ values rise significantly to 45.3 and 57.1 dB. In stark contrast, composites with low or no PEDOT content exhibit negligible SE across the THz range. A comparative summary of GHz and THz performance is provided in Table S2. Notably, the minor fluctuations observed in the high-frequency range (1.0–2.0 THz) arise from the combined effects of measurement limitations and the inherent frequency-dependent response of the heterogeneous composite. The signal-to-noise ratio of THz-TDS decreases at higher frequencies due to increased absorption and scattering, while interfacial polarization and microstructural heterogeneity may induce resonance-like features, consistent with previously reported THz shielding studies [50, 51]. These results collectively establish Al-Fe_3_O_4_/ANFs/PEDOT_3_ composite as a highly efficient, absorption-driven shield for THz frequencies, presenting compelling potential for next-generation electromagnetic stealth and secure communication technologies.

The underlying electromagnetic shielding and attenuation mechanism is schematically illustrated in Fig. 6g. Upon incident EMW irradiation, the initial impedance mismatch at the air-composite interface induces high-frequency surface currents, particularly along the conductive ANF network, leading to the partial reflection of incident waves [4, 52]. The transmitted portion of the EMW penetrates the gradient-structured interior, where it undergoes heterogeneous medium comprising Al-Fe_3_O_4_ nanosheets and the percolated ANF scaffold. These processes result in both constructive and destructive interference, effectively converting electromagnetic field energy into thermal energy through synergistic dielectric and magnetic loss mechanisms [32, 53]. For composites with PEDOT content below the electrical percolation threshold (Al-Fe_3_O_4_/ANFs and low PEDOT Al-Fe_3_O_4_/ANFs/PEDOT), the absence of a continuous conductive and magnetic network severely limits energy dissipation, resulting in low SE. As the PEDOT loading increases beyond the percolation threshold, a 3D interconnected conductive network forms. This network significantly enhances ohmic conduction, intensifies interfacial (Maxwell–Wagner–Sillars) polarization, and promotes field localization. The localized fields, in turn, activate magnetic resonances, including eddy-current losses and natural/exchange resonances [54, 55]. Simultaneously, PEDOT introduces a high density of dipolar sites and increases the overall matrix polarizability. At the numerous heterogeneous interfaces between PEDOT, ANFs, and Al-Fe_3_O_4_, oscillating dipoles efficiently dissipate energy through interfacial polarization and relaxation processes [9, 56]. Critically, the layered-gradient architecture imposes a deliberate spatial variation in both impedance and electrical conductivity across the film’s thickness. This graded design amplifies the trapping of incident EMWs and promotes multiple scattering events, significantly prolonging the propagation path and interaction time within the material. The outstanding broadband (GHz-THz) shielding performance of the Al-Fe_3_O_4_/ANFs/PEDOT_3_ composite thus originates from a sophisticated synergy of multiple mechanisms: initial surface reflection, intensive interfacial polarization, dipolar relaxation, pervasive multiple scattering, and coupled conductive-magnetic dissipation. This multi-faceted attenuation strategy positions the developed composite as a versatile and high-performance platform for advanced broadband electromagnetic management.

## Conclusion

In summary, we have successfully fabricated flexible layered Al-Fe_3_O_4_/ANFs/PEDOT composite films featuring a bioinspired, bamboo-like through-thickness gradient architecture via a scalable vacuum-assisted filtration strategy. This hierarchically programmed architecture consolidates multiple high-value functions including mechanical robustness, thermal stability, broadband GHz-THz EMW absorption, EMI shielding, and solvent-triggered intelligent actuation within a single, readily processable platform. At optimized PEDOT loadings, the composites exhibit exceptional electromagnetic attenuation, achieving a RL_min_ of − 56.6 dB at a practical thickness of 2.2 mm, while retaining notable mechanical compliance with a tensile strength up to 33.9 MPa. This combination underscores their suitability for conformal and wearable applications. The uniquely designed sawtooth-edged Al-Fe_3_O_4_ nanosheets enable precise tuning of complex electromagnetic parameters and, together with the ANF scaffold, furnish responsive sites that facilitate programmable, anisotropic actuation through directionally differential solvent swelling. Mechanistically, the bioinspired gradient architecture orchestrates multiscale energy dissipation, where dielectric loss, magnetic loss, interfacial polarization, and conductive loss are synergistically activated. The graded impedance profile at the air-film interface promotes efficient wave coupling and deep penetration. With increasing PEDOT content, a well-developed percolative conductive network synergizes with the embedded magnetic framework to deliver outstanding EMI shielding, yielding average total shielding effectiveness (SE_T_) values of 42.0 dB in the GHz band and 57.8 dB in the THz regime. In conclusion, the convergence of structural elegance (layered-gradient architecture), tailored materials chemistry (sawtooth Al-Fe_3_O_4_ nanosheets and gradient PEDOT), and scalable processing establishes a generalizable blueprint for multifunctional electromagnetic skins. The demonstrated synergy of broadband attenuation, mechanical integrity, and stimuli-responsive intelligence positions these composite films as promising candidates for advanced EMW management in fields ranging from aerospace and high-frequency communications to artificial intelligence hardware, soft robotics, and wearable electronics.

## Supplementary Information

Below is the link to the electronic supplementary material.

Supplementary file1 (MP4 1439 KB)

Supplementary file2 (DOCX 11131 KB)
