# Supplementary material for: Bioinspired Layered-Gradient Nanocomposites for Intelligent Electromagnetic Skins with GHz-THz Wave Absorption, Shielding, and Solvent-Driven Actuation
Source: Nanomicro Lett. 2026 Apr 24;18:344. doi: 10.1007/s40820-026-02202-y (PMC13109531; doi:10.1007/s40820-026-02202-y)
Supplement: Supplementary file 2 — Supplementary file2 (DOCX 11131 KB) [file 40820_2026_2202_MOESM2_ESM.docx]

Supporting Information for

Bioinspired Layered-Gradient Nanocomposites for Intelligent Electromagnetic Skins with GHz-THz Wave Absorption, Shielding, and Solvent-Driven Actuation

Xianyuan Liu^1^^#^, Yang Zhao^1#^, Yali Zhang^2^, Xuechun Cui^1^, Ying Xue^1^, Xianyong Lu^1,^ *, Junwei Gu^2,^ *

^1^State Key Laboratory of Bioinspired Interfacial Materials Science, School of Chemistry, Beihang University, Beijing 100191, P. R. China

^2^Shaanxi Key Laboratory of Macromolecular Science and Technology, School of Chemistry and Chemical Engineering, Northwestern Polytechnical University, Xi’an 710072, P. R. China

^#^ Xianyuan Liu and Yang Zhao contributed equally to this work.

*Corresponding authors. E-mail: [xylu@buaa.edu.cn](mailto:xylu@buaa.edu.cn) (Xianyong Lu); [nwpugjw@163.com](mailto:nwpugjw@163.com) & [gjw@nwpu.edu.cn](mailto:gjw@nwpu.edu.cn) (Junwei Gu)

**Supplementary Figures and Tables**


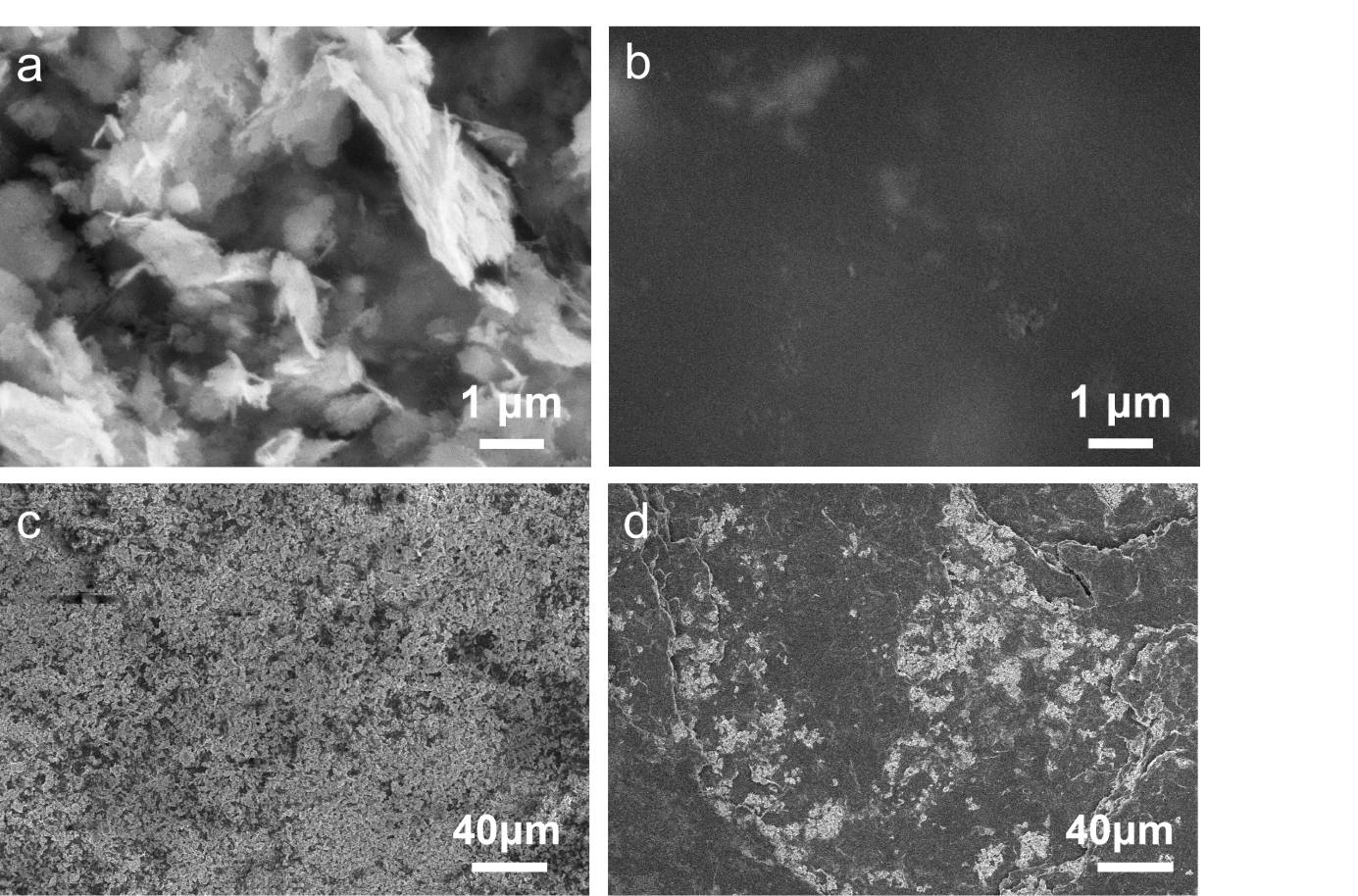


**Fig. S1** High-magnification SEM images of Al-Fe_3_O_4_/ANFs/PEDOT composite film. **a, c** Upper (top) surface and **b, d** lower (bottom) surface, highlighting the pronounced surface contrast imparted by the bamboo-inspired through-thickness gradient.

Consistent with the main text discussion, the high-magnification SEM observations directly reveal a substantially higher areal density of Al-Fe_3_O_4_ nanosheets on the lower (bottom) surface (Fig. S1b, d) than on the upper (top) surface (Fig. S1a, c). This surface-resolved contrast provides clear microstructural evidence for the filtration-induced gradient, namely a monotonic decrease in nanosheet coverage from the bottom toward the top of the composite film [S1].


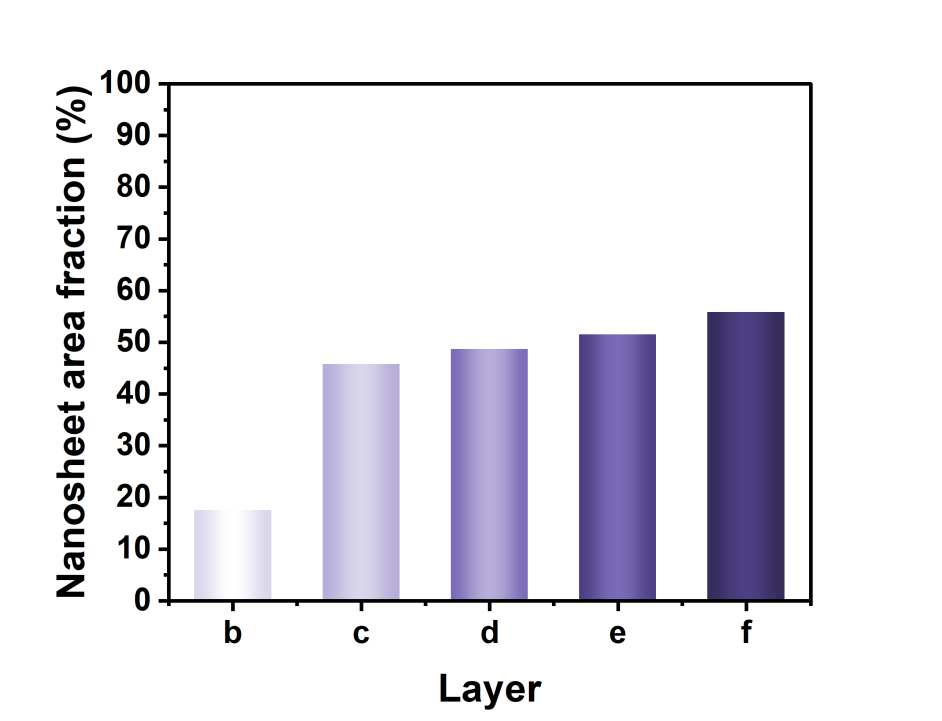


**Fig. S2** Areal fraction of Al-Fe_3_O_4_ nanosheets across five cryo-microtomed layers, quantitatively validating the through-thickness gradient architecture

Quantitative analysis of the nanosheet distribution along the film thickness was conducted using ImageJ based on the SEM images of the five cryo-sectioned layers (Fig. 3b-3f). To minimize processing-induced bias, all images were analyzed using an identical workflow with fixed parameters. Briefly, each image was converted to 8-bit grayscale and the contrast was uniformly adjusted to 0.35. A constant threshold window (120-255) was then applied to segment nanosheet-rich regions from the background, followed by particle analysis to determine the nanosheet areal fraction in each layer. The resulting histogram (Fig. S2) shows a clear monotonic increase in nanosheet coverage from the top to the bottom layer, providing quantitative confirmation of the well-defined through-thickness gradient structure in the composite film.


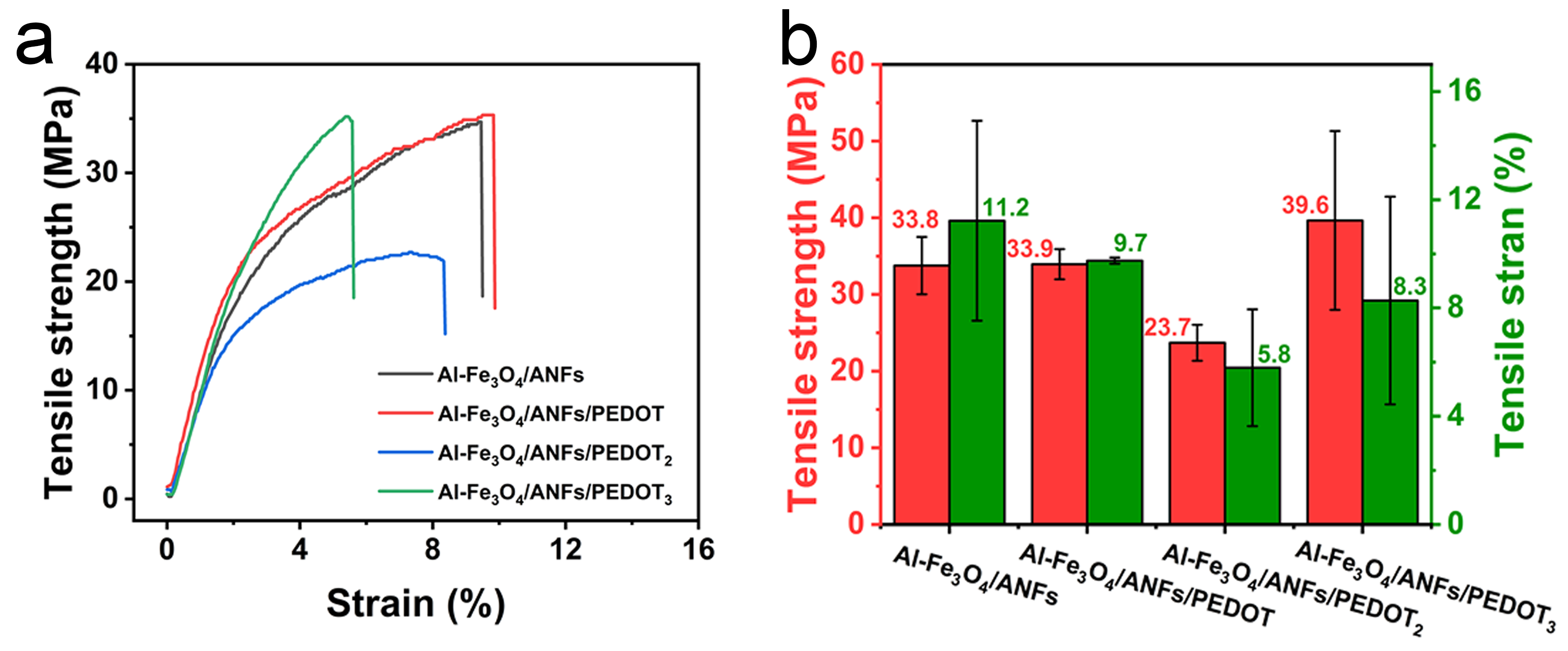


**Fig. S3** Mechanical properties of Al-Fe_3_O_4_/ANFs/PEDOT composite films. **a** Representative tensile stress-strain curves and **b** Average tensile strengths and strains at break of the corresponding samples

The mechanical performance of the Al-Fe_3_O_4_/ANFs/PEDOT composite films arises from the coupled contributions of the percolated ANF scaffold and the PEDOT phase. At low PEDOT loadings, the continuous, hydrogen-bonded ANF network dominates load transfer, thereby dictating both tensile strength and elongation at break. With increasing PEDOT content, the composite progressively evolves toward a load-bearing regime, in which PEDOT and ANFs competitively regulate stress distribution and deformation pathways. Notably, initial PEDOT incorporation partially perturbs the ANF hydrogen-bonding network, leading to a reduction in both strength and ductility. Increasing the PEDOT fraction enhances the contribution of its resilient phase to stress bearing, primarily through conductive bridging and reinforced inter-fiber coupling. This effectively counterbalances the disturbance in the ANF architecture, resulting in a progressive recovery of mechanical properties. In summary, these results establish the PEDOT loading as a key parameter for precisely tuning the mechanical integrity of the layered-gradient composites, without compromising their multifunctional performance.


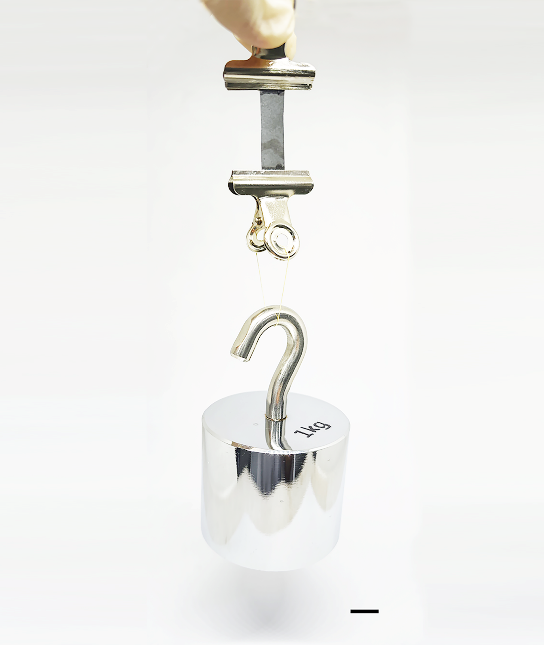


**Fig. S4** Optical photograph of the Al-Fe_3_O_4_/ANFs/PEDOT composite film (30 × 8 mm^2^) sustaining a uniaxial tensile load of 1000 g without failure (Scale bar: 1 cm)

This image corresponds directly to the mechanical robustness test in the text, visually confirming that the film maintains structural integrity under a substantial load (1000 g) without fracture or major deformation.


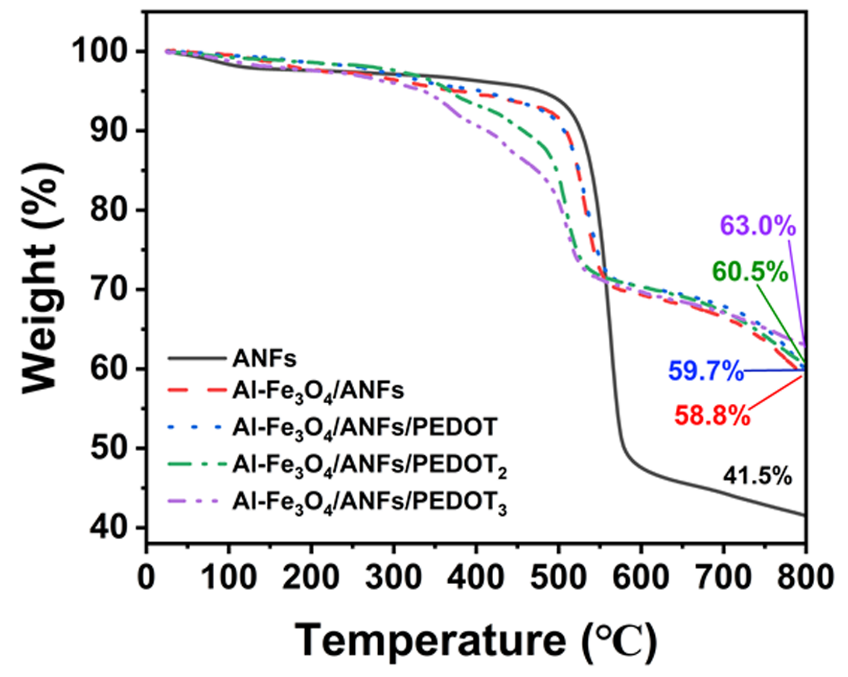


**Fig. S5** Thermogravimetric analysis (TGA) curves of the ANFs, Al-Fe_3_O_4_/ANFs, Al-Fe_3_O_4_/ANFs/PEDOT composite films

The TGA results reveal a significant enhancement in the thermal robustness of the films upon incorporation of Al-Fe_3_O_4_ nanosheets, attributable to the increased onset decomposition temperature relative to pristine ANFs. Furthermore, a progressive increase in PEDOT content elevates the residual char yield at 800 °C from 58.8% to 63.0%, underscoring a synergistic effect between the ceramic magnetic framework and the carbon-forming PEDOT phase in improving overall thermal endurance.


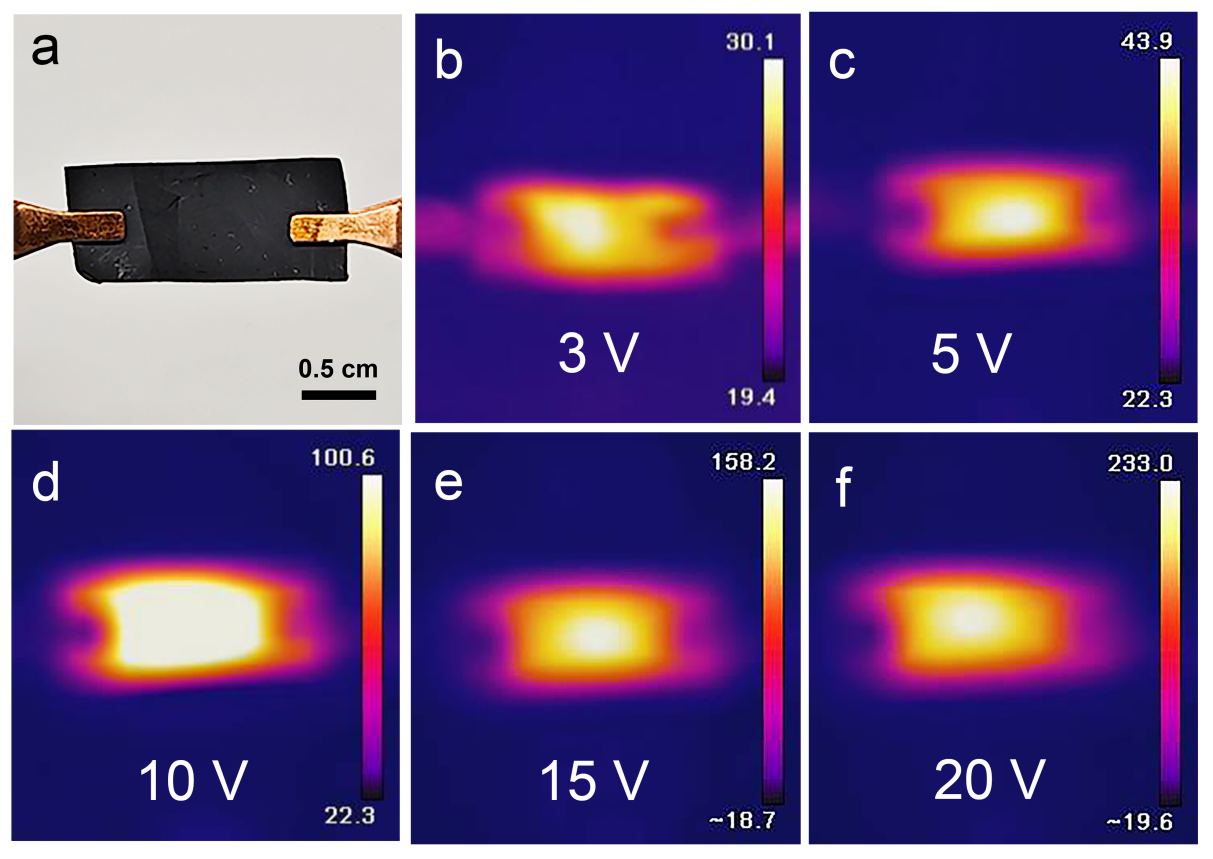


**Fig. S6** Joule-heating performance of Al-Fe_3_O_4_/ANFs/PEDOT_2_ composite films under applied voltages. **a** Optical image of the film and **b-f** Corresponding infrared thermal images acquired at different applied voltages, illustrating the voltage-dependent temperature rise and spatially uniform heat generation

Characterized by outstanding electrothermal performance, the Al‑Fe_3_O_4_/ANFs/PEDOT_2_ film reaches 233 °C at 20 V, exhibiting a nearly linear relationship between steady-state temperature and input voltage. The infrared thermographs **b-f** further attest to the attainment of high equilibrium temperatures and, importantly, reveal a homogeneous thermal field over the entire surface. These properties underscore the material's suitability for compact, high-power-density applications in thermal management and actuation.


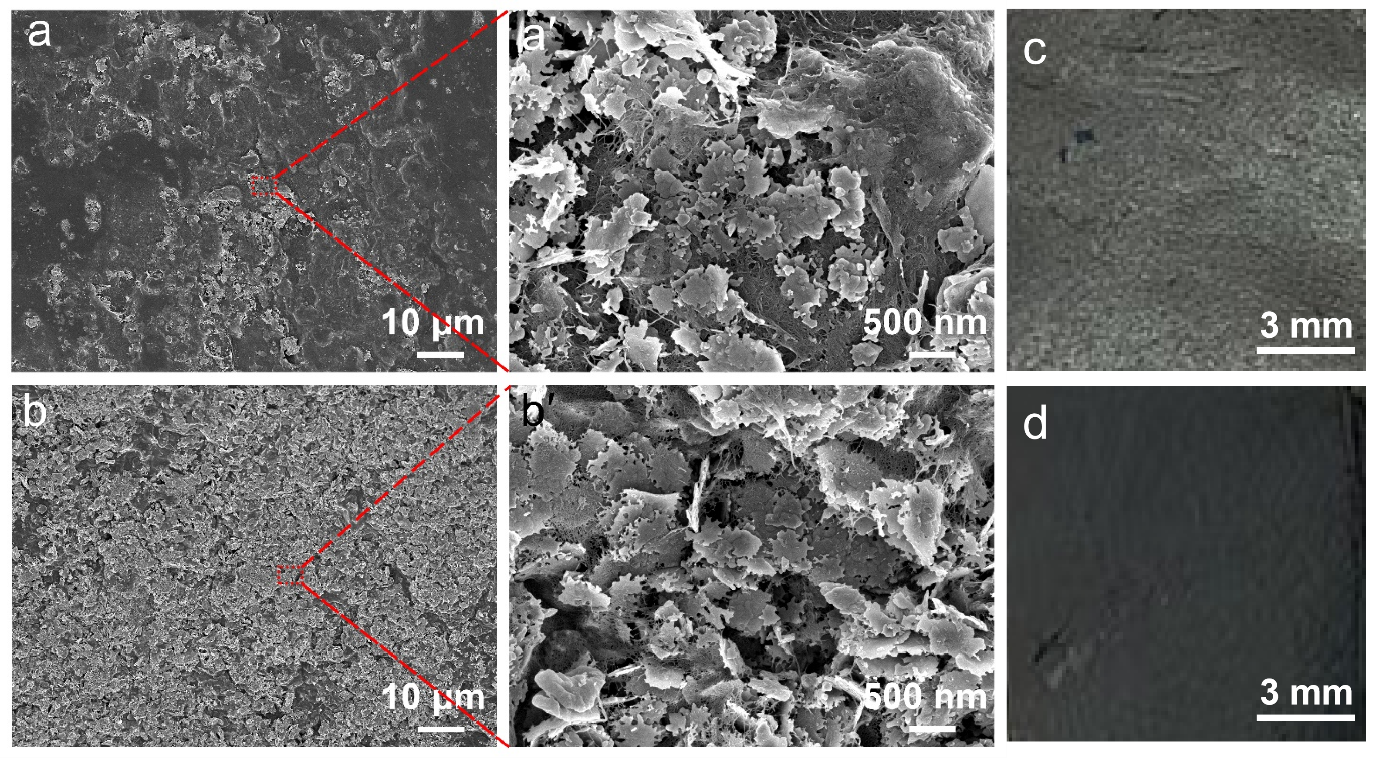


**Fig. S7** SEM images of Al-Fe_3_O_4_/ANFs/PEDOT_2_ composite film. (**a, a′**) Upper (top) surface and (**b, b′**) lower (bottom) surface. **c** Optical image of the top surface. **d** Optical image of the bottom surface

Despite the increased PEDOT loading, the Al-Fe_3_O_4_/ANFs/PEDOT_2_ film retains a well-defined gradient architecture through its thickness. As revealed by the surface morphology, the upper surface (Fig. S7a, a′) shows a relatively sparse distribution of Al-Fe_3_O_4_ nanosheets, in sharp contrast to the high-density accumulation on the lower surface (Fig. S7b, b′). Such a pronounced compositional contrast provides direct evidence for the successful fabrication of a bottom-enriched, layered-gradient structure enabled by the vacuum-assisted assembly strategy.


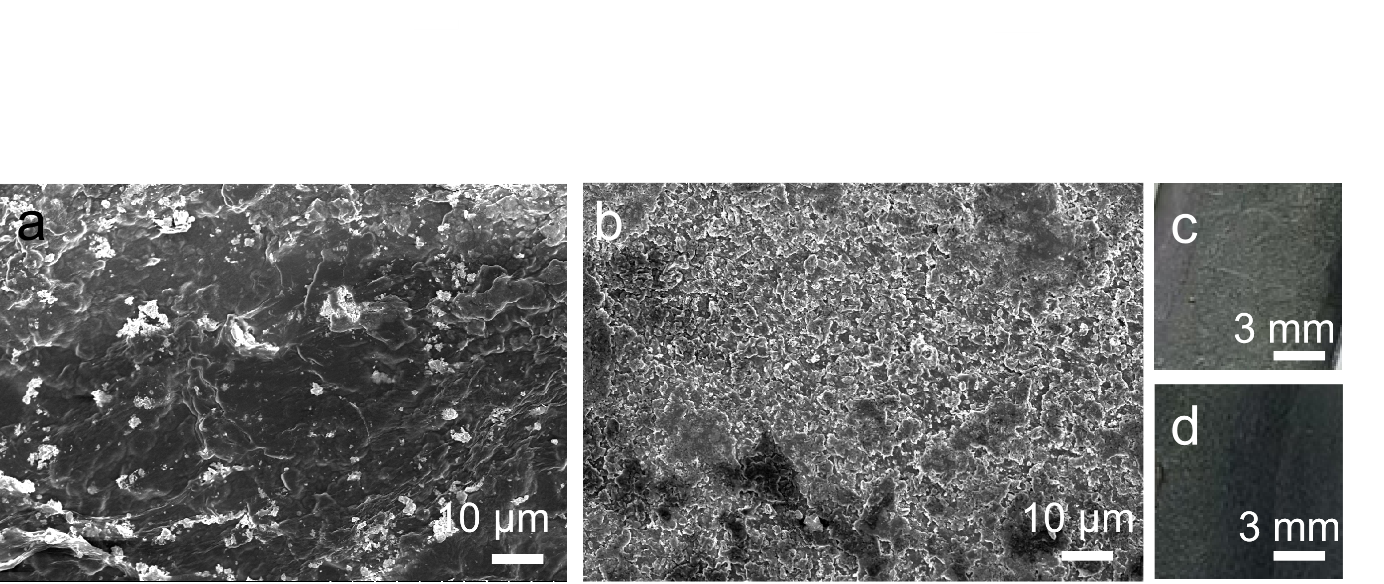


**Fig. S8** SEM images of Al-Fe_3_O_4_/ANFs/PEDOT_3_ composite film. (**a**) Upper (top) surface and (**b**) lower (bottom) surface. **c** Optical image of the top surface. **d** Optical image of the bottom surface


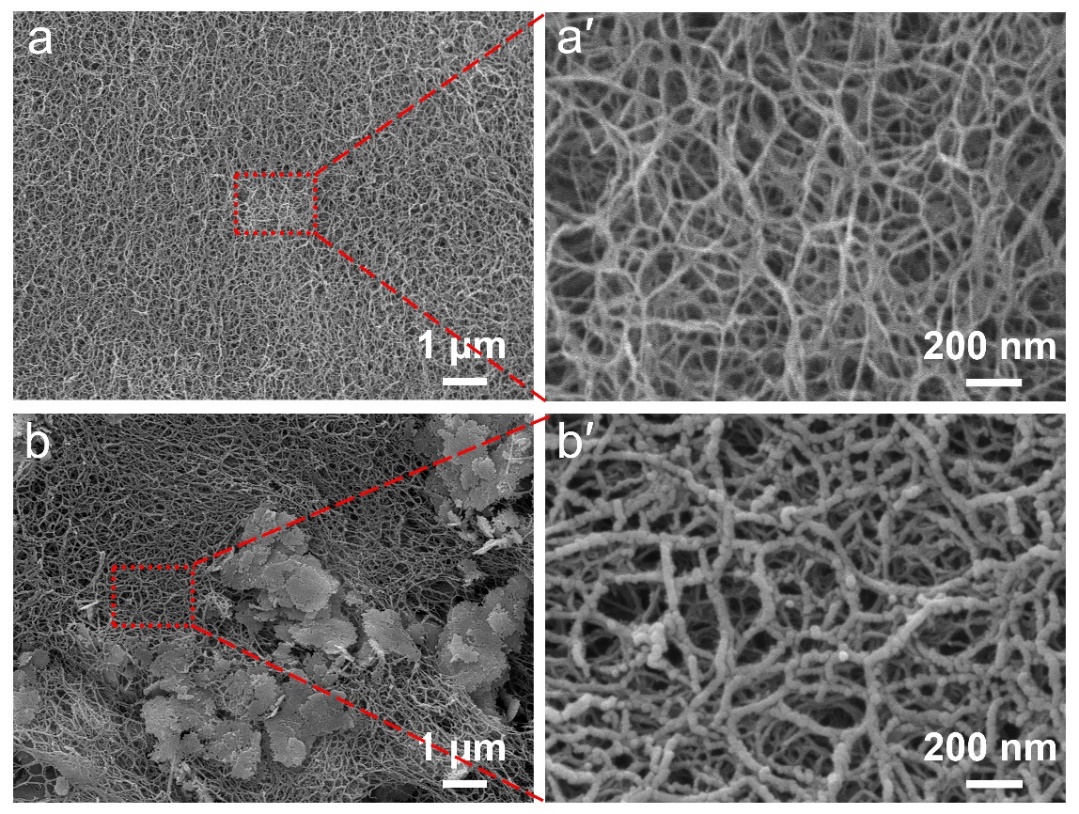


**Fig. S9** SEM images of aerogels prepared by supercritical CO_2_ drying. **a, a′** Surface morphology of the pristine ANF aerogel and **b, b′** Upper-surface morphology of the Al-Fe_3_O_4_/ANFs/PEDOT_2_ aerogel, highlighting preservation of the 3D ANFs scaffold and the conformal PEDOT coverage

The use of supercritical CO_2_ drying eliminates capillary stresses, thereby preserving the native porous morphology and revealing the intrinsic architectural features of the aerogels. In the composite aerogel (Fig. S9b, b′), the 3D ANF scaffold is retained and conformally coated with PEDOT, contrasting with the bare ANF structure (Fig. S9a, a′). As evidenced by the increase in average fiber diameter from 20 nm to 42 nm, a continuous core-shell nanofibrillar structure is achieved [S2]. This intimate interfacial contact facilitates an interconnected, low-resistance percolation network, which homogenizes current distribution and thermal flux to enable rapid, stable, and spatially uniform Joule heating. Collectively, this work demonstrates that synergistically integrating a porous 3D framework with a nanoscale conductive sheath constitutes a viable design principle for efficient electrothermal actuation in flexible multifunctional materials.


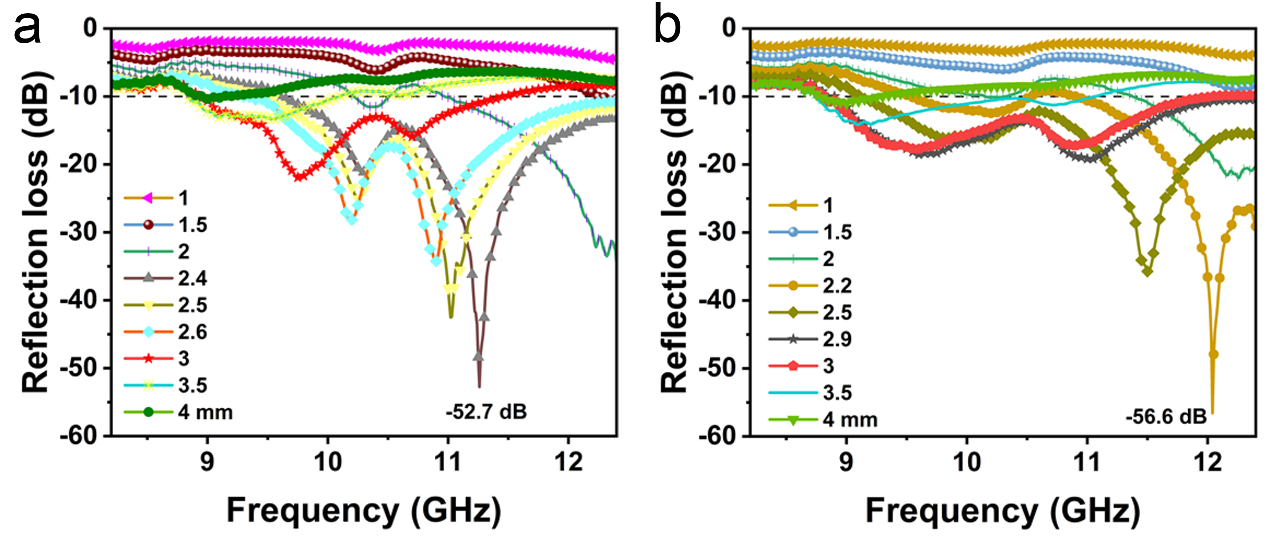

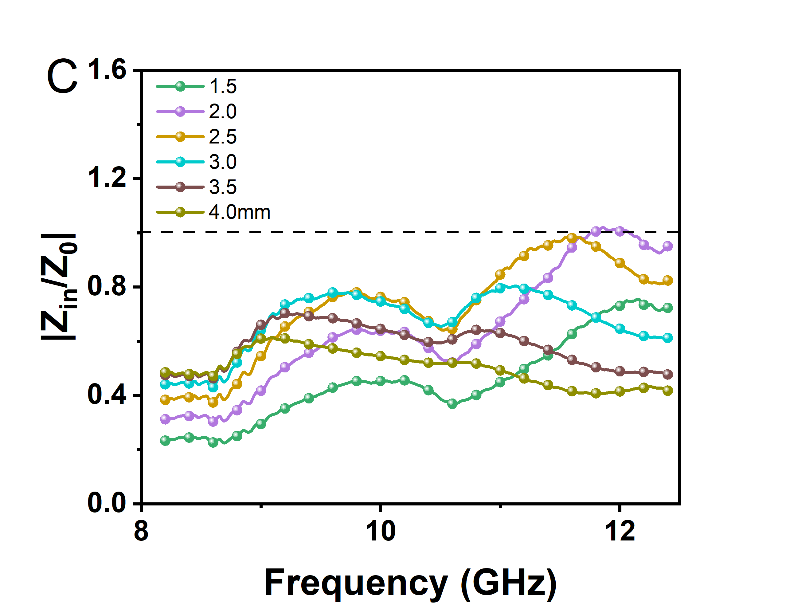


**Fig. S10** Two-dimensional reflection loss (RL) contour plots of **a** Al-Fe_3_O_4_/ANFs and **b** Al-Fe_3_O_4_/ANFs/PEDOT composites, highlighting the evolution of absorption performance as a function of frequency and absorber thickness. **c** The impedance matching curve of the Al-Fe_3_O_4_/ANFs/PEDOT film

The evolution of absorption performance with thickness and frequency is captured in the 2D RL contour maps (Fig. S10). The Al-Fe_3_O_4_/ANFs baseline exhibits an RL_min_ of -52.7 dB at 2.4 mm. With the incorporation of PEDOT, the composite achieves a superior RL_min_ of -56.6 dB at a thinner matching thickness of 2.2 mm, alongside a broader effective absorption bandwidth EAB of 3.5 GHz at 2.9 mm. This improved performance is achieved via the enhanced dielectric polarization and conductive losses contributed by PEDOT, highlighting the role of conductive-polymer engineering in optimizing the broadband electromagnetic absorption properties of the gradient composite.

**
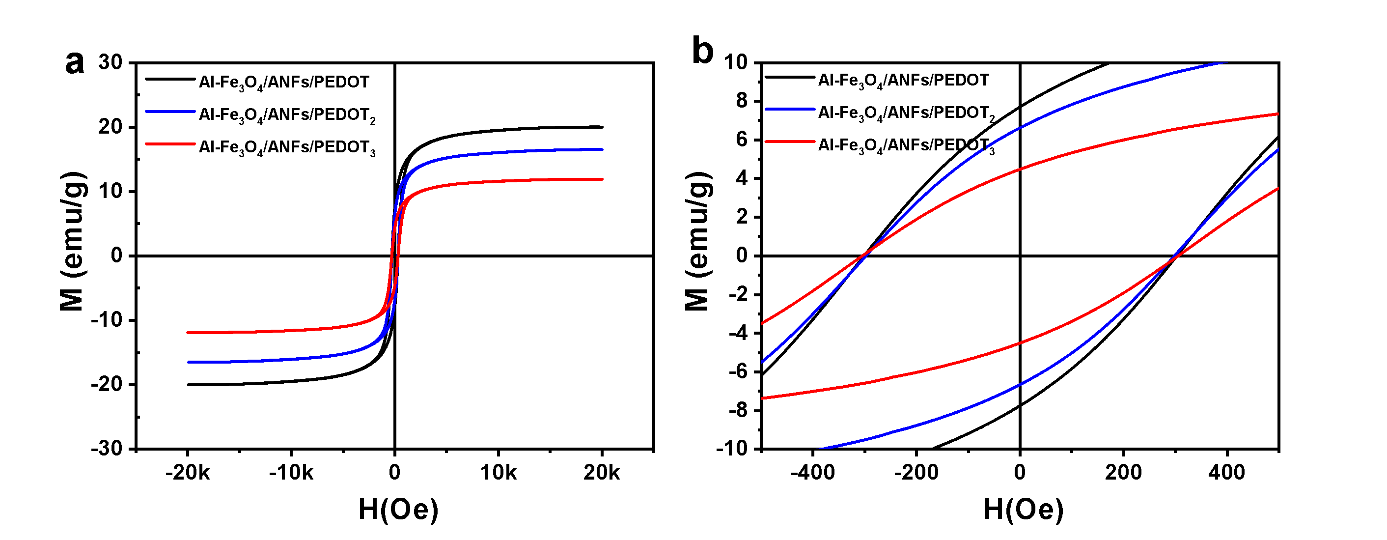
Fig. S11** Magnetic hysteresis loops of Al-Fe_3_O_4_/ANFs/PEDOT, Al-Fe_3_O_4_/ANFs/PEDOT_2_ and Al-Fe_3_O_4_/ANFs/PEDOT_3_ composite films measured at room temperature


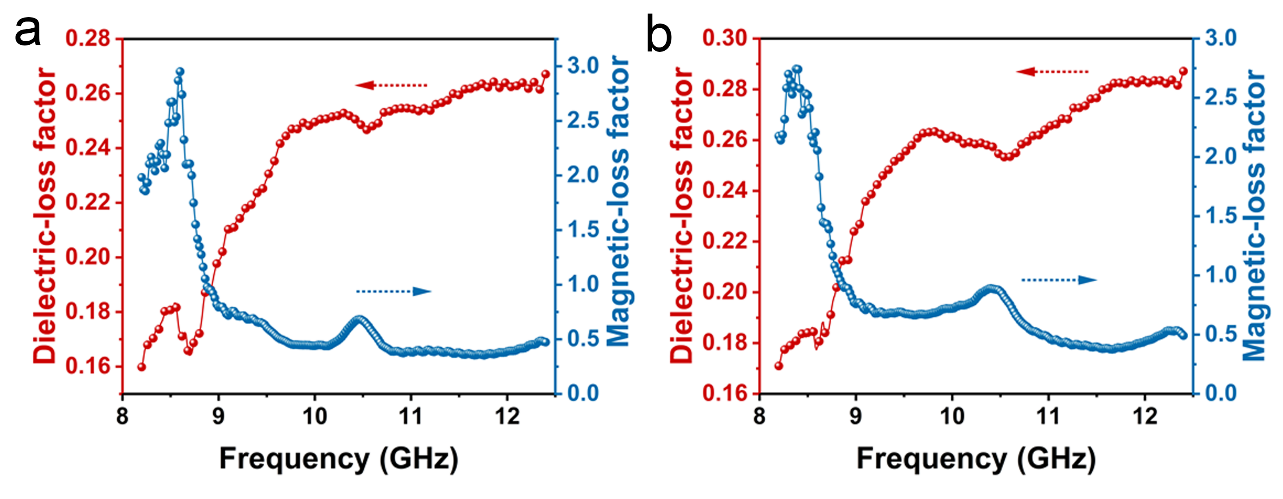


**Fig. S12** Dielectric and magnetic loss characteristics of the composites. Frequency-dependent dielectric loss factor (tan δ_ε_) and magnetic loss factor (tan δ_μ_) of (a) Al-Fe_3_O_4_/ANFs composites and (b) Al-Fe_3_O_4_/ANFs/PEDOT composites

For the Al-Fe_3_O_4_/ANFs/PEDOT_2_ and Al-Fe_3_O_4_/ANFs/PEDOT_3_ composites, the magnetic loss factor (tan δμ) remains higher than the dielectric loss factor (tan δε) within the 8-12 GHz range, identifying magnetic dissipation as the principal loss mechanism[3]. Importantly, when this magnetic dominance is coupled with the additional dielectric polarization and conductive losses from PEDOT, a synergistic interplay arises. This combination creates multichannel attenuation pathways that collaboratively broaden and intensify the overall dissipation of electromagnetic waves.


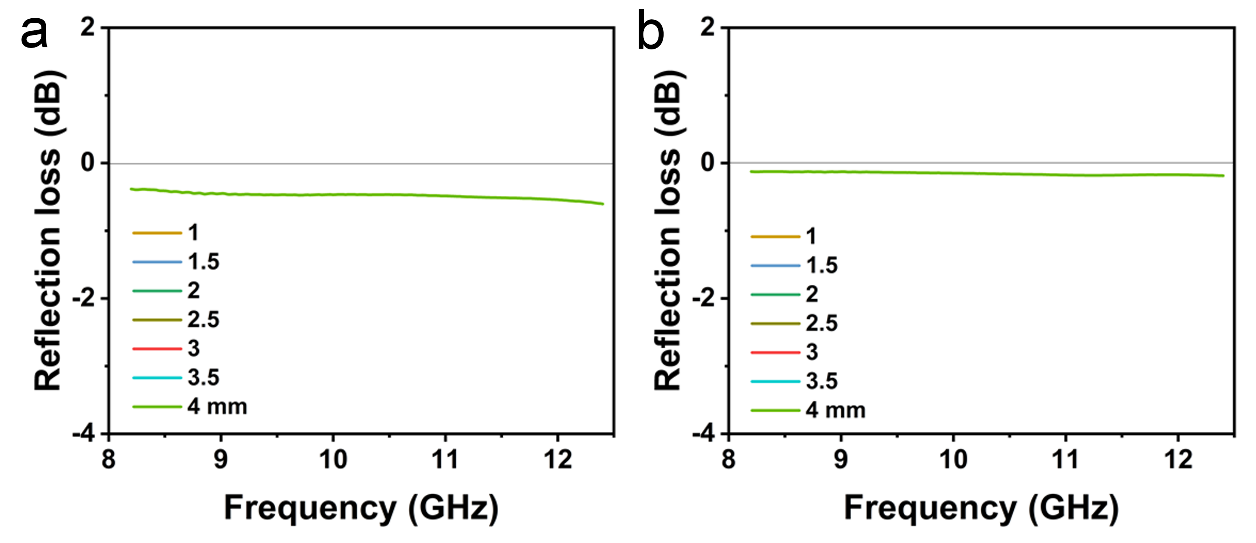


**Fig. S13** Reflection loss (RL) curves of **a** Al-Fe_3_O_4_/ANFs/PEDOT_2_ and **b** Al-Fe_3_O_4_/ANFs/PEDOT_3_ composites measured at different absorber thicknesses

The EMW absorption capability attenuates notably as the PEDOT content rises. For the Al-Fe_3_O_4_/ANFs/PEDOT_2_ composite, RL values approach 0 dB and the RL curves across different thicknesses converge, signaling weakened performance. This trend worsens with further PEDOT addition (Al-Fe_3_O_4_/ANFs/PEDOT_3_). The underlying mechanism is attributed to excessive conductivity, which severely mismatches the impedance at the air/absorber interface. Consequently, wave coupling is suppressed, and dissipation within the absorber is diminished. Collectively, this highlights a critical design principle: achieving optimal EMW absorption necessitates a precise compromise between constructing a conductive network and maintaining impedance matching, thereby ensuring the coexistence of multiple loss pathways.


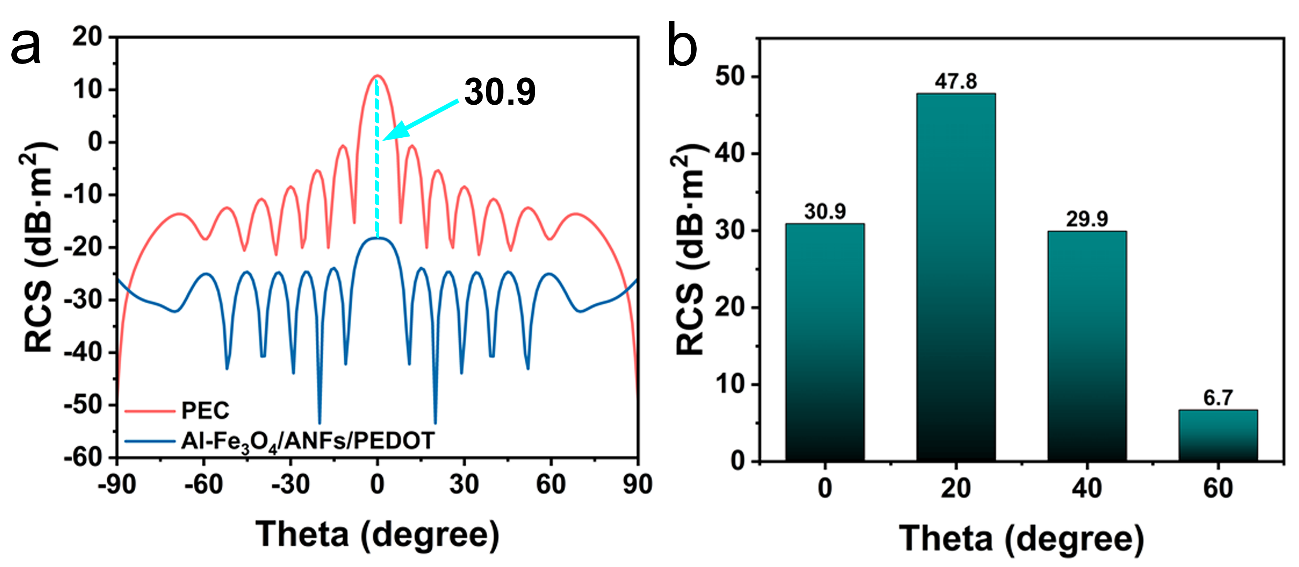


**Fig. S14** Simulated radar cross-section (RCS) performance. **a** RCS values of the modeled samples under selected incident angles. **b** Comparison of RCS reduction for a PEC substrate coated with Al-Fe_3_O_4_/ANFs/PEDOT versus the bare PEC substrate across different detection angles, highlighting the angle-tolerant backscatter suppression enabled by the absorber coating.

Simulation results confirm that the gradient-structured Al-Fe_3_O_4_/ANFs/PEDOT coating yields substantial RCS reduction over a broad span of incidence angles. At normal incidence (0°), it lowers the RCS from 12.7 dB·m^2^(bare PEC) to -18.2 dB·m^2^, a reduction of 30.9 dB·m^2^. The coating also displays remarkable angular stability, with RCS reductions of 47.8 dB·m^2^ at 20°, 29.9 dB·m² at 40°, and 6.7dB·m^2^ at 60°. This broadband, angle-insensitive performance, attributable to the gradient design, ensures effective suppression of both specular and diffuse scattering. The findings underscore the potential of this gradient film as a high-performance radar-absorbing coating for scenarios demanding reliable angular robustness.


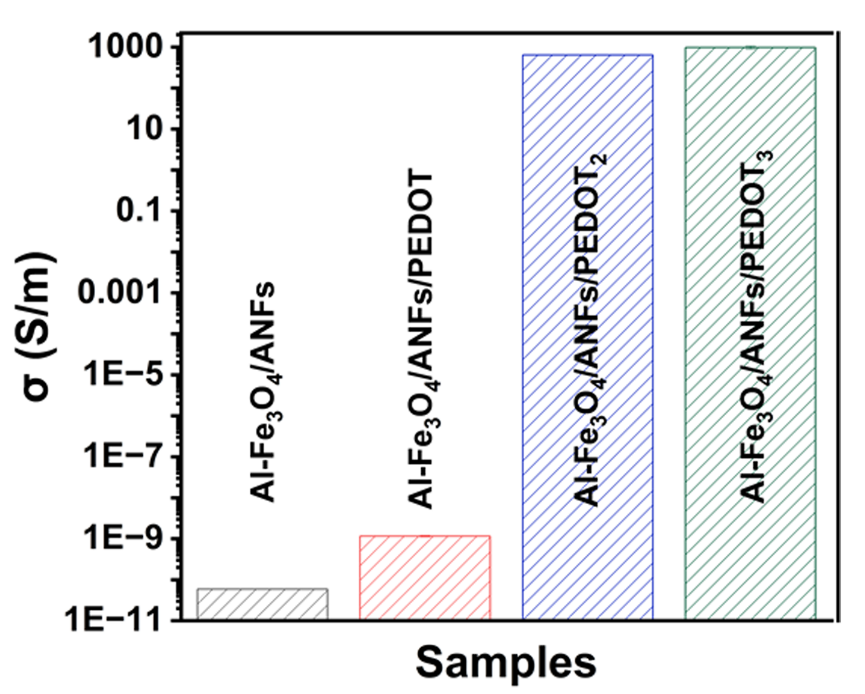


**Fig. S15** Electrical conductivity of Al-Fe_3_O_4_/ANFs/PEDOT composite films (and corresponding control samples) as a function of PEDOT content, highlighting the conductivity transition associated with conductive-network percolation

The electrical conductivity evolves from a near-insulating state (≈10⁻¹¹ S/m for the Al-Fe_3_O_4_/ANF baseline) to 975.721 S/m with increasing PEDOT content, spanning multiple orders of magnitude. This evolution originates from the development of a continuous PEDOT percolation network within the composite matrix, which establishes long-range, low-resistance charge transport pathways. This fundamental transition in conduction mechanism directly accounts for the observed shift in functionality: from an absorption-dominant EMW attenuator at low PEDOT fractions to an efficient EMI shield at higher PEDOT loadings a trend fully consistent with the enhanced shielding effectiveness (SE) documented in both the GHz and THz regimes.

**Table S1** Quantitative actuation performance of the composite film

| Parameter | Initial response time | Maximum bending angle | Average angular velocity during actuation phase | Recovery time | Average recovery speed | Complete cycle time |
| --- | --- | --- | --- | --- | --- | --- |
| Bottom surface | ~0.12 s | 238° | ~158°·s^-1^ | ~ 6.5 s | ~37°·s^-1^ | ~8 s |
| Top surface | ~0.22 s | 55° | ~37°·s^-1^ | ~ 5.5 s | — | ~7 s |

**Table S2** PEDOT content and electromagnetic wave absorption performance of the composite films (X-band, 8.2-12.4 GHz)

| Absorbers | Component | RL, min | | RL≤-10 dB | |
| --- | --- | --- | --- | --- | --- |
|  | PEDOT:PSS  (mL) | RL_min_  (dB) | Thickness  (mm) | EAB  (GHz) | Thickness  (mm) |
| Al-Fe_3_O_4_/ANFs | 0 | -52.7 | 2.4 | 3.1 | 2.6 |
| Al-Fe_3_O_4_/ANFs/PEDOT | 0.001 | -56.6 | 2.2 | 3.5 | 2.9 |

**Supplementary References**

1. X. Liu, J. Zhou, Y. Xue, X. Lu, Structural engineering of hierarchical magnetic/carbon nanocomposites *via* *in situ* growth for high-efficient electromagnetic wave absorption. Nano-Micro Lett. **16**(1), 174 (2024). <https://doi.org/10.1007/s40820-024-01396-3>
2. Y. Li, G. Ren, Z. Zhang, C. Teng, Y. Wu et al., A strong and highly flexible aramid nanofibers/PEDOT: PSS film for all-solid-state supercapacitors with superior cycling stability. J. Mater. Chem. A **4**(44), 17324–17332 (2016). <https://doi.org/10.1039/C6TA06981A>
3. N. Qu, H. Sun, Y. Sun, M. He, R. Xing et al., 2D/2D coupled MOF/Fe composite metamaterials enable robust ultra–broadband microwave absorption. Nat. Commun. **15**, 5642 (2024). <https://doi.org/10.1038/s41467-024-49762-4>
